# Nursing-Sensitive Outcomes among Patients Cared for in Paediatric Intensive Care Units: A Scoping Review

**DOI:** 10.3390/ijerph18189507

**Published:** 2021-09-09

**Authors:** Matteo Danielis, Adele Castellano, Elisa Mattiussi, Alvisa Palese

**Affiliations:** Department of Medical Sciences, School of Nursing, University of Udine, Viale Ungheria 20, 33100 Udine, Italy; adele.castellano1@gmail.com (A.C.); elisa.mattiussi@uniud.it (E.M.); alvisa.palese@uniud.it (A.P.)

**Keywords:** critically ill patient, paediatrics, paediatric intensive care unit, nursing-sensitive outcomes, scoping review

## Abstract

Measuring the effectiveness of nursing interventions in intensive care units has been established as a priority. However, little is reported about the paediatric population. The aims of this study were (a) to map the state of the art of the science in the field of nursing-sensitive outcomes (NSOs) in paediatric intensive care units (PICUs) and (b) to identify all reported NSOs documented to date in PICUs by also describing their metrics. A scoping review was conducted by following the framework proposed by Arksey and O’Malley. Fifty-eight articles were included. Publications were mainly authored in the United States and Canada (*n* = 28, 48.3%), and the majority (*n* = 30, 51.7%) had an observational design. A total of 46 NSOs were documented. The most reported were related to the clinical (*n* = 83), followed by safety (*n* = 41) and functional (*n* = 18) domains. Regarding their metrics, the majority of NSOs were measured in their occurrence using quantitative single measures, and a few validated tools were used to a lesser extent. No NSOs were reported in the perceptual domain. Nursing care of critically ill children encompasses three levels: improvement in clinical performance, as measured by clinical outcomes; assurance of patient care safety, as measured by safety outcomes; and promotion of fundamental care needs, as measured by functional outcomes. Perceptual outcomes deserve to be explored.

## 1. Introduction

Paediatric intensive care units (PICUs) provide comprehensive care to children suffering from both acute life-threatening conditions (e.g., traumatic brain injuries; surgery, e.g., congenital heart disease) and chronic diseases (respiratory, e.g., asthma; metabolic, e.g., diabetes) [1]. The number of PICU beds per 100,000 individuals ranges from 2.7 per 100,000 children in the United Kingdom [2] to 8.0 per 100,000 children in the United States [1]. PICUs in the United Kingdom admit around 20,000 children/year, with 96.5% of them being discharged alive between 2017 and 2019 [3], whereas U.S. PICUs’ bed growth exceeded paediatric population growth over 15 years [1], with a total of 5908 beds and an observed PICU mortality of 2.31% [4].

Alongside mortality rates, many studies have focused their attention on other outcomes affecting the PICU length of stay (LOS). A 2018 retrospective cohort study on paediatric patients with a LOS of 14 days or longer identified that 52 (22%) acquired one or two nosocomial infections [5]. Out of the 16 bloodstream infections, three were catheter-related bloodstream infections (CRBSIs); moreover, 12 (5%) children acquired gastrointestinal complications, 25 (10%) developed deep vein thrombosis, and 12 (5%) developed pressure ulcers [5]. Furthermore, children were documented to develop other complications during their PICU stay, such as ventilator-associated pneumonias (VAPs; 53.1%) and catheter-associated urinary tract infections (CAUTIs; 28.1%) [6].

In this context, nursing care has been documented to prevent complications and to optimise outcomes [7]. Consequently, a broader set of health outcomes is currently used to measure the quality of nursing care, linking nursing interventions to outcomes for patients [8], also termed in the literature ‘nursing-sensitive outcomes’ (NSOs) [9]. However, while NSOs have been largely conceptualised, used, and measured among adult patients who are both chronically [10] and critically [11] ill, no published or ongoing reviews (e.g., registered in the PROSPERO database) have been produced on this topic on the paediatric population. Some studies focusing on PICU settings have reported mortality and adverse effects as NSOs predominantly linked to nursing skill-mix and staffing ratios [12]. A 2016 survey also identified that higher levels of nursing education and experience were significantly associated with fewer postoperative complications, in-hospital mortality, and failure to rescue rate [13]. As a consequence, to our best knowledge, no secondary studies have been performed to date to summarise the available literature on NSOs in the paediatric ICU population. Having such studies might have multiple concrete implications. First, for researchers, having a map of the state of the science in the field, also regarding the outcomes investigated to date and thus accepted by the scientific community to be linked with nursing care, might support the defining of conceptual frameworks of their research according to the evidence available, and the availability of a summary of evidence might also help to overcome the limitations of current research by identifying new lines of outcome-related research. For clinical nurses, such evidence might provide support in deciding which outcomes merit increased surveillance and documentation, whereas for educators, it might offer support in deciding the core elements of the curriculum, both in undergraduate and postgraduate education. Lastly, for nurse managers, this contribution might address issues in measuring nursing care as well as in improving its quality. According to these multiple implications, a comprehensive overview on nursing outcomes in PICUs is called for [14]. Specifically, this review is aimed at mapping out the current literature on NSOs in PICUs by exploring all outcomes conceptualised to date.

## 2. Material and Methods

### 2.1. Study Aims

The aim was twofold: (a) to map the state of the art of the science in the field of NSO in paediatric ICUs and (b) to identify all reported NSOs documented to date in PICUs by also describing their metrics.

### 2.2. Study Design

A scoping review was conducted by following the framework proposed by Arksey and O’Malley [15]. Accordingly, the following five steps were adopted: (a) establishing the research question; (b) identifying relevant studies; (c) selecting the studies; (d) charting the data; and (e) collating, summarising, and reporting the findings. The Preferred Reporting Items for Systematic reviews and Meta-Analysis extension—Scoping Reviews (PRISMA-ScR) flow diagram guidance was used [16] (Table S1).

### 2.3. Establishing the Research Question

The population, concept, and context model (PCC model) was used [17]. According to the PCC model, three distinct elements have been identified: paediatric critically ill patients, including individuals <18 years old experiencing life-threatening conditions [1]; nursing-sensitive outcome(s), as the actual change measured in patients’ health status linked to the nursing care received [9]; and PICUs, as the physical space designated for the treatment of paediatric patients who require intensified, comprehensive monitoring and critical care [18]. As a consequence, two research questions were addressed: (a) What is the state of the science in the field of NSOs in the PICU setting? and (b) What are the PICUs’ NSOs that have been conceived and measured to date in the available literature?

### 2.4. Identifying Relevant Studies

The search strategy was developed through the use of Medical Subject Headings (MeSH) together with free-text keywords in different combinations, resulting in four search strings for Medline (PubMed) and three for the Cumulative Index to Nursing and Allied Health Literature (CINAHL) (Table 1). Database searching was performed in April 2020 (last search date 30th April 2020). No time limits for identifying relevant studies were chosen in order to gain a comprehensive view by an inclusive approach. In addition, the reference lists of included articles were manually evaluated to identify any additional and relevant publications. Duplicate studies, if any, were eliminated through manual inspection.

### 2.5. Study Selection

The first author (M.D., male, PhD student, nurse educator, expert in intensive care) and the second author (A.C., female, nursing student) reviewed titles and abstracts using the inclusion criteria (Table 2), supervised by the last author (A.P., female, associate professor in nursing science). In accordance with the method described by Arksey and O’Malley [15], we did not perform quality appraisal of the included studies.

### 2.6. Charting the Data

To extract data from the included studies, we developed a grid form in a Microsoft Excel spreadsheet. The following data were extracted: (a) general information (authors, year of publication, country of the study); (b) main features (study design, aims and setting, sample characteristics); and (c) a description of the reported nursing-sensitive outcome(s), metrics, interventions implemented, and key findings. Secondary sources (e.g., literature reviews) were also encompassed in the data analysis as single studies. The data extraction table is available in Table S2. Data extraction was performed individually by two authors (M.D., A.C.) and then compared to eliminate inconsistencies.

### 2.7. Collating, Summarising, and Reporting the Results

Two authors (M.D., A.C.) reviewed and discussed the findings. On the basis of the research questions, we performed a synthesis of the literature by summarising the main characteristics of the included studies (e.g., overall number of studies, years of publication, countries where studies were conducted). Then, the charted NSOs were grouped into seven sub-domains (healthcare-associated infections, critical incidents, general health, goal assessment and monitoring, physical dimension, psychosocial dimensions, and experience of being in intensive care) according to the recent literature in the field [11] established in the context of adult ICUs, in order to increase the likelihood of comparison across settings and to accumulate evidence in the field of the frameworks available. Moreover, NSOs were then categorised in four domains (safety, clinical, functional, and perceptual) according to Doran [9]. Specifically, NSOs were classified as (a) safety (e.g., pressure ulcers), (b) clinical (e.g., readmission rate to PICU in 48 h), (c) functional (e.g., oral health), and (d) perceptive outcomes (e.g., patient’s experience). In addition, each NSO was described by briefly reporting the metrics (e.g., tool used) documented in the study. 

All of these phases resulted from authors’ independent work followed by discussions and agreement.

## 3. Results

### 3.1. The State of the Art of NSOs Research in PICU Settings

**Figure 1:**
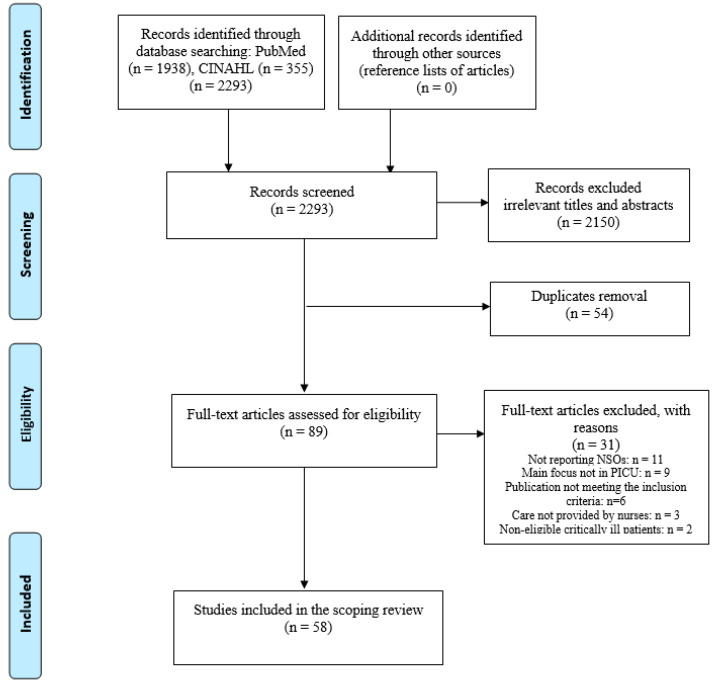
Preferred reporting items for systematic reviews and meta-analyses flow diagram [16]. Note: CINAHL: Cumulative Index to Nursing and Allied Health Literature; NSOs: nursing sensitive outcomes; PICU: paediatric intensive care unit.

Of the 2293 studies initially identified, 58 met the inclusion criteria (Figure 1). No studies published earlier than 1999 emerged; most of the included studies were published from 2010 to 2020 (*n* = 50, 86.2%). The majority (*n* = 28, 48.3%) of the studies were performed in the United States and Canada, followed by Europe (*n* = 10, 17.2%), the Middle East (*n* = 7, 12.1%), Australia and New Zealand (*n* = 6, 10.3%), Asia (*n* = 4, 6.9%), and Central and South America (*n* = 3, 5.2%). Studies were mainly conducted in general PICUs (*n* = 53, 91.4%), while the remaining studies were conducted in specialised units, such as cardiovascular or medical and surgical PICUs (*n* = 5, 8.6%). 

Fifty studies were primary studies and eight were reviews. Among the primary studies, 30 (51.7%) were observational in design and 20 (34.5%) were experimental or quasi-experimental. A summary of the characteristics of the main study is displayed in Table 3, whereas a full description is reported in Table 1.

### 3.2. NSOs and Metrics Documented to Date in PICUs

The 58 selected studies reported 143 NSOs, categorised into 46 outcomes. Table 4 summarises the outcomes in their domains and sub-domains. According to Doran’s classification [9], the most reported outcomes were related to the clinical domain (*n* = 83), followed by those belonging to the patient safety (*n* = 41) and functional (*n* = 18) domains. No NSOs emerged in the perceptual domain as defined by Doran [9].

With regard to the safety domain, within the healthcare-associated infections sub-domain (*n* = 23 NSOs), the most commonly reported outcome was VAPs (*n* = 8), followed by healthcare-associated infections (*n* = 5) and CRBSIs, which were reported in four publications. Lastly, CAUTIs and central line-associated bloodstream infections (CLABSIs) were each reported in three studies. Within the critical incidents sub-domain (*n* = 18 NSOs), the most reported outcome was pressure ulcers (*n* = 8), followed by unplanned/accidental extubations (*n* = 2) and mobilisation-related adverse events (*n* = 2). In addition, aspiration events, post-pyloric tube placement events, radiation exposures, hematomas, peripheral intravenous catheter (PIVC) failures, and overdrawn blood volume were each reported once.

Studies evaluating the clinical domain documented NSOs in their general health (*n* = 59 NSOs) and goal assessment and monitoring (*n* = 24 NSOs) sub-domains. The most reported outcomes in the general health dimension were PICU LOS (*n* = 18), mortality (*n* = 16), and duration of mechanical ventilation (*n* = 10). Hospital LOS and morbidity were each measured in four studies, while readmission rate to PICU within 48 h, weaning duration, and reintubation were measured in two. Moreover, weaning failure was reported in one study. Within the goal assessment and monitoring dimension, pain/distress and sedation and agitation level were the most reported outcomes in three studies, followed by vomiting, occurrence of iatrogenic withdrawal, and PIVC dwell time, each in two publications. Other outcomes (e.g., ventilator-free days, post-extubation stridor) were each reported once.

Some studies also documented the functional domain, including the physical (*n* = 12 NSOs) and the psychosocial (*n* = 6 NSOs) dimensions. Within the physical sub-domain, nutritional status (*n* = 6) was the most reported outcome, followed by oral health (*n* = 3), hypoglycaemia (*n* = 2), and mobilisation (*n* = 1). Within the psychosocial sub-domain, delirium was the most reported outcome in four studies, while cognitive status and noise pollution were each reported once.

As for metrics, the majority of NSOs were measured by their occurrence with quantitative single measures (e.g., prevalence/incidence rates of infections, number of events of vomiting, days with a specific symptom). On the other side, validated tools emerged to a lesser extent. In the case of pain, the Critical Care Pain Observation Tool (CPOT); the Numeric Rating Scale (NRS); the Face, Legs, Activity, Cry, Consolability (FLACC) scale; the Individualised Numeric Rating Scale; and the Wong-Baker Faces Pain Scale were reported as having been used [19], whereas the State Behavioural Scale was reported as having been adopted to assess sedation and agitation level. With regard to delirium, the Cornell Assessment of Paediatric Delirium (CAPD) and the PreSchool Confusion Assessment Method for the ICU (psCAM-ICU) were documented [20], while the Glasgow Come Scale and the Full Outline of Un Responsiveness (FOUR) methodologies were adopted to assess the cognitive status of children [21]. Lastly, to predict mortality, the Paediatric Risk of Mortality (PRISM) score, the Paediatric Index of Mortality (PIM) 2 score, the Paediatric Index of Mortality (PIM) 3 score, and the Sequential Organ Failure Assessment (SOFA) were reported as having been developed [22]. These tools and measures, as summarised in Table 4, can be useful for clinical nurses, nurse educators, and managers to address their practice.

## 4. Discussion

To the best of our knowledge, this is the first review synthesising the existing literature on NSOs in PICU settings. Periodic mapping of the state of the art in a research field might inform nurses in their different roles (clinical, educational, managerial) regarding the knowledge available in order to inform directions to undertake in their daily practice. Moreover, the map might also inform researchers in identifying gaps as well as in detecting frameworks and trends used in a specific field.

### 4.1. The State of the Art of NSO Research in the PICU Setting

A total of 58 studies emerged from this review, mainly primary studies, most of them having been published in the last 10 years, suggesting a relatively recent interest in nursing outcomes in the paediatric critical care field. In a recent review of studies up to February 2019 regarding adult ICU settings, 112 studies emerged [11], with a similar impulse in the last 10 years—suggesting that in the critical care field both primary and secondary studies investigating NSOs in the field are increasing.

In general, the majority of studies were conducted in the United States and Europe, whereas data from low- and middle-income countries—where there is a higher percentage of hospitalised paediatric patients compared to other countries—are sparse. Because most of the articles identified originated in the United States and Europe, caution is required when generalising from our findings to the paediatric population worldwide. Results from the studies under review might reflect the different availability of PICU beds across countries and differences in the conditions to access necessary medications, supplies, and equipment [23]. A global approach in summarising the literature may be useful in critical care research to globally evaluate paediatric services and to apply real beneficial care for critically ill children [24]. However, in the tendency of some hospitals to aggregate paediatric patients in adult ICUs, data from the paediatric population may fail to emerge; therefore, researchers are encouraged to underline the different population groups when reporting their ICU findings to allow continuing scrutiny of this research field, despite the different models of care across the world that might combine paediatric and adult patients.

Some data emerged from experimental studies (*n* = 20, 33.9%), while the majority of studies were observational. Moreover, in the context of adult ICU research field, a relatively small number of experimental studies (*n* = 26, 23.2%) emerged [11]. In general, experimental study designs have been documented to be limited in the nursing field [25]—with a call to action to increase studies assessing effectiveness. However, the limited studies that also emerged in the PICU field can be interpreted as a difficulty to identify specific nursing care interventions with a strong relationship with patients’ outcomes because children in PICUs typically receive care from a multidisciplinary team.

### 4.2. NSOs and Metrics Documented to Date in PICUs

The analysis of 58 studies allowed for the identification of 46 outcomes as capable of detecting the contribution of nursing care in PICUs. The heterogeneity of critically ill paediatric patients (e.g., age, size, medications) suggests that paediatric critical care requires specific measures for both quality and safety [26]. Better understanding of patients’ outcomes might help clinical nurses and researchers to make appropriate choices about patients’ care by improving decision making and preventing complications [13]. Moreover, the set of NSOs that emerged from this review can be used at the research level to develop a PICU minimum data set [27] to allow for evaluation of the nursing care offered and its outcomes by increasing the accuracy of data collected.

Concerning outcomes that emerged, the majority of studies evaluated clinical NSOs, suggesting that the main goal for paediatric patients is to have them rapidly recovered from critical illness. Moreover, the PICU LOS (*n* = 18) was the most frequently investigated outcome, followed by mortality (*n* = 16) and the length of mechanical ventilation (*n* = 10). These outcomes are crucial measures of resource use and healthcare facility performance [28]. Moreover, clinical outcomes, as well as data on patient complications (e.g., readmissions, reintubations), are easily accessible through patients’ charts and hospital discharge databases [9]. In addition, the broader set of outcomes under the goal assessment and monitoring sub-domain (e.g., pain and distress, sedations, and agitation level) makes the nursing involvement in patient care explicit, thus suggesting their essential role in improving clinical performance and safety by detecting children at risk of clinical worsening [29].

As for frequency, studies reported safety outcomes as the second main domain, indicating, hence, that health-care-associated infections and critical incidents are a tangible concern in the PICU setting. Healthcare professionals have moved from a culture of acceptance, in which complications and adverse effects were expected, to a point where these harms are no longer tolerable and are preventable in many cases [30], both for the individual and for the collective costs [31]. Several studies have also shown that some negative outcomes (e.g., nosocomial infections) lead to substantial additional LOS and morbidity [5], thus strengthening the side effects of hospitalisation that also merit consideration by nurses. According to our findings, the set of outcomes under the safety domain should be strictly monitored by paediatric critical care nurses in order to ensure a robust culture of safety and to prevent complications, or at least to report them in a homogeneous manner across settings, thus allowing comparison and benchmarking.

We found that functional outcomes were less often explored. Within the physical sub-domain, nutritional status was the most reported outcome. Unbalanced macronutrient intake during childhood critical illness has been associated with increased morbidity (e.g., prolonged mechanical ventilation, increased infection rates) as well as increased mortality [32]. Nurse-led feeding protocols in PICUs, along with timely nutritional assessments, should be encouraged to ensure adequate energy intake and to prevent delays in nutritional support. In addition, both physical and psychosocial outcomes—which are those closer to the fundamentals of nursing care (e.g., mobilisation, cognitive status) [33]—need to be fully explored with the aim of portraying the specific contribution of PICU nurses, as there are few documented experiences that would make it possible to draw any conclusions in this regard.

Finally, perceptual outcomes have been neglected in the available literature. We find it troubling that there is a lack of research examining children’s own perspectives regarding their experiences of being in an intensive care unit, despite evidence that paediatric patients wish to receive medical information and are able to contribute to health-related discussions [34,35]. In practice, nurses have been reported to consider the relatives’ experiences as a proxy for the assessment of the perceptual domain [34]. Moreover, paediatric nurses have reported the risk of a child’s autonomy being undermined by the parents as an ethical issue in the bedside care of children [35]. Perceptual outcomes (e.g., emotions, health-care-induced anxiety, satisfaction with care) should be more often considered in the future, alongside the need for providing holistic care to the patient. 

The outcomes we found were reported under different approaches and metrics. Interestingly, the majority of outcomes were quantitative and expressed single indicators (prevalence, incidence) over a period, and in a given population. In a previous scoping review including studies regarding adult ICU patients [11], a greater occurrence of validated tools emerged, and less often direct indicators. This peculiarity might be interpreted under different angles: (a) as a result of a lack of tools in the paediatric field to measure outcomes in intervention studies, suggesting therefore a call for action—mainly in those dimensions not evaluable directly with physiological (e.g., cardiac rate frequency) or performance (e.g., LOS) measures, and (b) as a concrete attempt to establish single and direct measures in a field of research, rendering easy and accurate comparisons across settings on the main issues (e.g., prevalence/incidence rates, such as events per 1000 ventilator-days or VAP per 1000 intubated patients). The continuous assessment of this research field with future scoping reviews might help in understanding trends in this field; however, despite the substantial homogeneity across NSOs in metrics as compared to previous research in other settings [11], a consensus is necessary to establish common definitions and metrics for each NSO.

### 4.3. Limitations

There are several limitations for the present scoping review. Firstly, whilst a wide-ranging search strategy and an inclusive approach were adopted, some eligible studies might have been missed. Moreover, a potential selection bias may have been introduced given that only two databases were explored; furthermore, to develop a comprehensive map, secondary studies were also included. 

Secondly, we used available frameworks to categorise the 46 outcomes that emerged from studies. This inductive/deductive approach [11] might have introduced biases in detecting outcomes; on the other side, the frameworks might have been imposed in a field of research where no previous summary of the studies available has been produced. Although the first limitation was prevented by our conducting independent extractions and then agreeing upon the findings, the second suggests that future researchers should assess the validity of the framework used in the paediatric ICU field. Thirdly, according to the methods established in conducting the scoping review [15], no qualitative evaluation of the studies included was performed, therefore limiting the possibility to assess the quality of the research methods used in this field of investigation. Fourthly, studies were analysed in detail, reporting the age (months, years) of the population included in each study, while the map of the NSOs and the metrics have been summarised as a whole without differentiating each according to the age. This decision was made given the limited studies that emerged; however, in future reviews, a subgroup analysis is encouraged.

## 5. Conclusions

Exploring nursing outcomes in paediatric settings is a field under rapid development. The analysis of our results—based on existing literature—indicates that 46 NSOs can actually contribute to detect the quality of care in PICUs. While some outcomes are well documented (e.g., PICU LOS, mortality, length of mechanical ventilation, VAPs, pressure ulcers), which might help to address decisions in managing critical care resources, other outcomes remain less explored (e.g., physical and psychosocial dimensions) or never explored (e.g., the experience of being in intensive care).

According to the findings, nursing care of critically ill children encompasses three levels: (1) improvement of clinical performance, as measured by clinical outcomes (e.g., pain and distress); (2) assurance of patient care safety, as measured by safety outcomes (e.g., unplanned/accidental extubations); and (3) promotion of fundamental care needs, as measured by functional outcomes (e.g., nutritional status). Perceptual outcomes, which are closely related to the lived experience of children being cared for in PICUs, deserve to be studied. Regarding the metrics, several quantitative single and direct measures, in forms of indicators, were reported as having been used, while limited validated tools have been considered to date among studies examining the efficacy of some nursing interventions.

From a clinical point of view, nurses might use the set of NSOs emerged from this review when deciding which outcomes merit being reported in documentation. From an educational level point of view, nurse educators might issue them as a blueprint to select the contents of the curriculum, both in undergraduate and postgraduate education. Moreover, for what concerns policy making, nurse managers might use the results to address priorities in nursing documentation and to set indicators to evaluate the nursing quality of care over time, benchmarking the data also across settings. Lastly, researchers might use the outcomes emerged when they design intervention studies to accumulate evidence by also advancing the frameworks used to categorise NSOs and to cover the gaps identified in this field of research.

## Figures and Tables

**Table 1:** Literature search strategy for MEDLINE (PubMed) and CINAHL, conducted in April 2020.

Search	Query
MEDLINE (PubMed)	
1	“Outcome Assessment, Health Care” [Mesh] AND “Intensive Care Units, Paediatric” [Mesh] AND “Paediatric Nursing” [Mesh]
2	“Critical Care Outcomes” [Mesh] AND “Intensive Care Units, Paediatric” [Mesh] AND “Paediatric Nursing” [Mesh]
3	“Outcomes Measures” AND “Intensive Care Units, Paediatric” [Mesh] AND “Paediatric Nursing” [Mesh]
4	“Quality of Health Care” [Mesh] AND “Critical Care Outcomes” [Mesh] AND “Paediatric Nursing” [Mesh]
CINAHL	
1	Paediatric intensive care unit + outcomes + nursing
2	Intensive care unit, paediatric + quality of health care + paediatric nursing
3	Intensive care unit, paediatric + patient outcomes + nursing
Limited to English language and paediatric population (<18 years)	

Note: CINAHL, Cumulative Index to Nursing and Allied Health Literature.

**Table 2:** Inclusion criteria.

Item	Inclusion
Language	English language
Publication time	Any
Population	Paediatric population (<18 years of age)
Setting	Paediatric intensive care unit
Study design	All quantitative and qualitative study designs, as well as secondary studies based on all types of reviews
Study focus	Concerning paediatric patients admitted and cared for in PICU settings

Note: PICU = paediatric intensive care unit.

**Table 3:** Summary of the main study characteristics (*n* = 58).

Study Characteristic	Number of Studies (*n* = 58) *n*. (%)
**Year of publication**	
January 1999 to December 2009	8 (13.8)
January 2010 to April 2020	50 (86.2)
**Continent**	
US and Canada	28 (48.3)
Europe	10 (17.2)
Middle East	7 (12.1)
Australia and New Zealand	6 (10.3)
Asia	4 (6.9)
Central and South America	3 (5.2)
**Setting (PICU type)**	
General	53 (91.4)
Specialised (e.g., cardiac intensive care)	5 (8.6)
**Study design**	
Observational	30 (51.7)
Experimental and quasi-experimental	20 (34.5)
Literature review	8 (13.8)

Note: PICU = paediatric intensive care unit; US = United States.

**Table 4:** Overview of identified NSOs in PICUs (*n* = 142), according to their domain, sub-domain, and metrics used.

Outcome Domain (n.) [9]	Outcome Sub-Domain (n.) [11]	Reported Outcome(s) and n. of Publications Including Each Specific Outcome (n.)	Metrics of NSOs and Brief Description
**Safety (41)**	Healthcare- associated infections (23)	VAP (8)	Prevalence/incidence rate (e.g., events per 1000 ventilator-days, VAP per 1000 intubated patients)
		Healthcare-associated infections (e.g., colonisation of the oropharynx) (5)	Prevalence/incidence rate (e.g., nosocomial infection rate per 1000 PICU-days)
		CRBSI (4)	Prevalence/incidence rate (e.g., events per 1000 catheter-days)
		CAUTI (3)	Prevalence/incidence rate (e.g., events per 1000 device-days)
		CLABSI (3)	Prevalence/incidence rate (e.g., events per 1000 line-days)
	Critical incidents (18)	Pressure ulcers (8)	Prevalence/incidence rate (e.g., events per 100 PICU-days)
		Unplanned/accidental extubations (2)	Prevalence/incidence rate (e.g., events per 100 ventilator-days)
		Mobilisation-related adverse events (2)	Number of events (e.g., line removal)
		Aspiration events (1)	Number of events
		Postpyloric tube placement events (1)	Number of events (e.g., oropharyngeal bleeding)
		Radiation exposures (1)	Number of events
		Haematoma (1)	Prevalence/incidence rate
		PIVC failures (e.g., occlusion) (1)	Prevalence/incidence rate
		Blood volume overdrawn (1)	Millilitre/kilogram per PICU-days
**Clinical (83)**	General health (59)	PICU LOS (18)	Number of PICU-days
		Mortality (16)	Mortality rate; 90-days mortality; paediatric risk of mortality (PRISM) score; paediatric index of mortality (PIM) 2 score; paediatric index of mortality (PIM) 3 score
		Duration of mechanical ventilation (10)	Number of intermittent mandatory ventilation days
		Hospital LOS (4)	Number of PICU-days
		Morbidity (4)	Diseased state (e.g., severity of organ dysfunction)
		Readmission rate to PICU in 48 h (2)	Prevalence/incidence rate
		Weaning duration (2)	Hours
		Reintubation (2)	Prevalence/incidence rate
		Weaning failure (1)	Prevalence/incidence rate
	Goal assessment and monitoring (24)	Pain and distress (3)	Critical Care Pain Observation Tool; Numeric Rating Scale
		Sedation and agitation level (3)	State Behavioural Scale
		Vomiting (2)	Number of events
		Occurrence of iatrogenic withdrawal (2)	Number of events
		Ventilator-free days (2)	Days
		PIVC dwell time (2)	Catheterisation-days; PIVC-hours
		Vasoactive-free days (1)	Days
		PICU-free days (1)	Days
		Diarrhoea (1)	Prevalence/incidence rates
		Post-extubation stridor (1)	Prevalence/incidence rates
		Clinical deterioration (1)	Paediatric early warning systems scores
		Nasojejunal tube (1)	Number of successful placements
		Changes in intracranial pressure (1)	Measurement in mm/hg
		Changes in mean arterial pressure and cerebral perfusion pressure (1)	Measurement in mm/hg
		Change of PIVC dressing (1)	Prevalence/incidence rates
		Incidence of red-blood cells transfusion (1)	Prevalence/incidence rates
**Functional (18)**	Physical dimension (12)	Nutritional status (6)	Prevalence/incidence rates; adequate feeding (90–110% of required kcal/die); underfeeding (<90% of required kcal/die); overfeeding (>110% of required kcal/die)
		Oral health (3)	Oral assessment scale; culturing oropharyngeal flora; amount of mucositis; presence of dental plaque accumulation
		Hypoglycaemia (2)	Prevalence/incidence rate
		Mobilisation (1)	Protocol adherence
	Psychosocial dimension (6)	Delirium (4)	Cornell Assessment of Paediatric Delirium Tool; Preschool Confusion Assessment Method for the ICU
		Cognitive status (1)	Glasgow Coma Scale
		Noise pollution (1)	Hourly decibel readings
**Perceptual (-)**	Experience of being in PICU (-)	(-)	(-)

Note: PICU = paediatric intensive care unit; NR = non-reported; VAP = ventilator-associated pneumonia; CRBSI = catheter-related bloodstream infection; CAUTI = catheter-associated urinary tract infection; CLABSI = central line-associated bloodstream infection; PIVC = peripheral intravenous catheter; LOS = length of stay; Kcal = kilocalorie.

## Supplementary Materials

The following are available online at https://www.mdpi.com/article/10.3390/ijerph18189507/s1, Table S1: Preferred Reporting Items for Systematic reviews and Meta-Analyses extension for Scoping Reviews (PRISMA-ScR) Checklist; Table S2: Data extraction process.
