# Supplementary material for: Nursing-Sensitive Outcomes among Patients Cared for in Paediatric Intensive Care Units: A Scoping Review
Source: Int J Environ Res Public Health. 2021 Sep 9;18(18):9507. doi: 10.3390/ijerph18189507 (PMC8468044; doi:10.3390/ijerph18189507)
Supplement: Supplementary file 1 [file ijerph-18-09507-s001.zip › ijerph-1359623-supplementary.pdf]

Table S1. Preferred Reporting Items for Systematic reviews and Meta-Analyses extension for Scoping Reviews (PRISMA-ScR) Checklist.

SECTION	ITEM	PRISMA-ScR CHECKLIST ITEM	REPORTED ON PAGE #
**TITLE**			
Title	1	Identify the report as a scoping review.	1
**ABSTRACT**			
Structured summary	2	Provide a structured summary that includes (as applicable): background, objectives, eligibility criteria, sources of evidence, charting methods, results, and conclusions that relate to the review questions and objectives.	1
**INTRODUCTION**			
Rationale	3	Describe the rationale for the review in the context of what is already known. Explain why the review questions/objectives lend themselves to a scoping review approach.	2–3
Objectives	4	Provide an explicit statement of the questions and objectives being addressed with reference to their key elements (e.g., population or participants, concepts, and context) or other relevant key elements used to conceptualise the review questions and/or objectives.	2–3
**METHODS**			
Protocol and registration	5	Indicate whether a review protocol exists; state if and where it can be accessed (e.g., a web address); and if available, provide registration information, including the registration number.	Not reported
Eligibility criteria	6	Specify characteristics of the sources of evidence used as	4

SECTION	ITEM	PRISMA-ScR CHECKLIST ITEM	REPORTED ON PAGE #
		eligibility criteria (e.g., years considered, language, and publication status), and provide a rationale.	
Information sources*	7	Describe all information sources in the search (e.g., databases with dates of coverage and contact with authors to identify additional sources), as well as the date the most recent search was executed.	4
Search	8	Present the full electronic search strategy for at least 1 database, including any limits used, such that it could be repeated.	4, Table 1
Selection of sources of evidence †	9	State the process for selecting sources of evidence (i.e., screening and eligibility) included in the scoping review.	4, Table 2
Data charting process ‡	10	Describe the methods of charting data from the included sources of evidence (e.g., calibrated forms or forms that have been tested by the team before their use, and whether data charting was done independently or in duplicate) and any processes for obtaining and confirming data from investigators.	4, Figure 1
Data items	11	List and define all variables for which data were sought and any assumptions and simplifications made.	4
Critical appraisal of individual sources of evidence §	12	If done, provide a rationale for conducting a critical appraisal of included sources of evidence; describe the methods used and how this information was used	Not reported

SECTION	ITEM	PRISMA-ScR CHECKLIST ITEM	REPORTED ON PAGE #
		in any data synthesis (if appropriate).	
Synthesis of results	13	Describe the methods of handling and summarising the data that were charted.	5
**RESULTS**			
Selection of sources of evidence	14	Provide numbers of sources of evidence screened, assessed for eligibility, and included in the review, with reasons for exclusions at each stage, ideally using a flow diagram.	5–7
Characteristics of sources of evidence	15	For each source of evidence, present characteristics for which data were charted and provide the citations.	5–7, Table 3
Critical appraisal within sources of evidence	16	If done, present data on critical appraisal of included sources of evidence (see item 12).	Not reported
Results of individual sources of evidence	17	For each included source of evidence, present the relevant data that were charted that relate to the review questions and objectives.	5–7
Synthesis of results	18	Summarise and/or present the charting results as they relate to the review questions and objectives.	5–7, Table 4
**DISCUSSION**			
Summary of evidence	19	Summarise the main results (including an overview of concepts, themes, and types of evidence available), link to the review questions and objectives, and consider the relevance to key groups.	7–9
Limitations	20	Discuss the limitations of the scoping review process.	10
Conclusions	21	Provide a general interpretation of the results with respect to the review questions and	11

SECTION	ITEM	PRISMA-ScR CHECKLIST ITEM	REPORTED ON PAGE #
		objectives, as well as potential implications and/or next steps.	
**FUNDING**			
Funding	22	Describe sources of funding for the included sources of evidence, as well as sources of funding for the scoping review. Describe the role of the funders of the scoping review.	Not reported

JBI = Joanna Briggs Institute; PRISMA-ScR = Preferred Reporting Items for Systematic reviews and Meta-Analyses extension for Scoping Reviews. * Where *sources of evidence* (see second footnote) are compiled from, such as bibliographic databases, social media platforms, and websites. † A more inclusive/heterogeneous term used to account for the different types of evidence or data sources (e.g., quantitative and/or qualitative research, expert opinion, and policy documents) that may be eligible in a scoping review as opposed to only studies. This is not to be confused with *information sources* (see first footnote). ‡ The frameworks by Arksey and O'Malley (6) and Levac and colleagues (7) and the JBI guidance (4, 5) refer to the process of data extraction in a scoping review as data charting. § The process of systematically examining research evidence to assess its validity, results, and relevance before using it to inform a decision. This term is used for items 12 and 19 instead of "risk of bias" (which is more applicable to systematic reviews of interventions) to include and acknowledge the various sources of evidence that may be used in a scoping review (e.g., quantitative and/or qualitative research, expert opinion, and policy document).

Table S2. Data extraction process (n=58 studies)

First author, year, country, setting	Design	Aim(s)	Participants' profile	Nursing sensitive outcomes (metrics)	Nursing care intervention(s)	Key findings
**Duzkaya et al, 2015** Turkey, PICU	Randomised controlled trial	To compare and evaluate the effects of intermittent feeding through a nasogastric catheter with continuous feeding through a nasoduodenal catheter in preventing VAP in the PICU	n=40 children, n1=20 NG, n2=20 ND, aged between 1 month and 18 years	VAP (VAP per 1,000 ventilator-day)	Intermittent feeding through a nasogastric catheter - continuous feeding through a nasoduodenal catheter	The incidence of paediatric VAP was 15%. The rate of VAP in patients who were ND feeding was 10%, whereas the rate of VAP in patients who had NG feeding was 20%. No statistically significant difference was observed between the ND- and NG-feeding patients. ND feeding helped to reduce the incidence of VAP
**Rosenthal et al, 2012** Argentina, PICU	Randomised controlled trial	To examine the effect of the INICC's multidimensional infection control program comprising a bundle of infection control interventions, education, outcome surveillance, process surveillance, feedback on VAP rates, and performance feedback on infection control practices don reducing the incidence of VAP in the PICUs of 8 INICC member hospitals in 5 developing countries, and to advance the study of the specific impact of this preventive strategy on VAP rates in limited-resource countries	n=4,339 patients, hospitalized for a total of 29,209 days in 8 PICUs, with a total 15,106 (MV)-days. Patient characteristics were similar in the baseline and intervention phases. Age between 7-21 months	VAP (VAP per 1,000 mechanical-ventilator-day)<br>CAUTI (CAUTI per 1,000 device-day)<br>CLABSI (NR)<br>microorganisms' profile<br>bacterial resistance<br>LOS-PICU (days)<br>Mortality (PICU mortality rate)	A period of active surveillance without the implementation of the multidimensional infection control program (phase 1) to be compared with rates of VAP after implementing such a program (phase 2), which included the following: bundle of infection control interventions, education, outcome surveillance, process surveillance, feedback on VAP rates, and performance feedback on infection control practice	During the baseline period, there was recorded a total of 5,212 MV-days; during implementation of the intervention bundle, there was recorded 9,894 MV-days. The VAP rate was 11.7 per 1,000 MV-days during the baseline period and 8.1 per 1,000 MV-days during the intervention period, demonstrating a 31% reduction in VAP rate. Implementation of the International Nosocomial Infection Control Consortium's multidimensional program was associated with a significant reduction in VAP rate in PICUs
**Horn et al, 2003** Australia, PICU	Randomised controlled trial	To examine the relationship between two gastric feeding regimens, continuous and intermittent, and children's tolerance	n=45 children, assigned to either the continuous (n=22) or the intermittent (n=23) gastric feeding groups	N° of stools (per day)<br>Diarrhoea and vomiting (Prevalence and/or incidence rate)<br>The prevalence of diarrhoea and vomiting was calculated by using three different methods: n. of participants with one on more episodes, n. of episodes per participant, percentage of days	Administration of NE and monitoring of effects	The number of stools/day and the prevalence of diarrhoea and vomiting did not differ significantly between the groups. The mean number of bowel movement per 24-hour period was 1.3 for the continuously fed group and 1.6 for the intermittently fed group. Further gastric feeding studies and the development of enteral feeding guidelines for critically ill children are needed
**Duyndam et al, 2020** Netherlands,	Prospective study (pre-post)	To find out if the use of a nurse-driven ventilation	n=424 patients, aged from 0 to 18 years;	Durations of IMV (DOV) (IMV-days)	Nurse-driven weaning protocol. The protocol	The median duration of ventilation in the pre-test and post-test periods was 42.5 h vs. 44.5 h.

PICU		weaning protocol in a PICU can shorten the duration of mechanical ventilation	n1=212 pre-test n2=212 post-test	LOS-PICU (days)<br>Reintubation (Prevalence and/or incidence rate)<br>Time of extubation (NR)	includes a two-step algorithm: first, weaning of oxygen and PEEP and second, choosing a support mode (volume support in case of PRVC and pressure support in case of PC) and gradually weaning off pressure or volume	In the post-test period, the PICU stay was non significantly shorter: 5.5 days vs 7 days (pre-test period). The reintubation rate in pre-test and post-test periods was 5% vs. 7%. Implementation of a nurse-driven weaning protocol did not result in a significantly shorter duration of invasive mechanical ventilation but was safe and successful. The reintubation rate did not significantly increase compared with usual care
**Gurgueira et al, 2005**<br>Brazil,<br>MICU<br>SICU	Retrospective cohort study	To evaluate the effect of parenteral nutrition (PN) and enteral nutrition (EN) on in PICU mortality before and after a continuous education program in nutrition support that leads to implementation of a nutrition support team (NST)	n=323 patients, between zero and 2 years of age, with a LOS over 72 hours, middle age: 4 months	Mortality (Mortality rate)<br>LOS-PICU (days)<br>DOV (IMV-days)	A continuous education program in nutrition support that leads to implementation of a nutrition support team (NST)	Progressive increase was observed in EN use, median values for which were 25% in period 1 and rose to 67% by period 5 in medical patients; there was no significant difference in surgical patients. A reduction was observed in PN use; in period 1 medians were 73% and 69% for medical and surgical patients respectively and decreased to 0% in period 5 for both groups. There was significant reduction in-PICU mortality rate during period 4 and period 5 among medical patients. The predictive factors for risk of death were duration of lung mechanical ventilation 7 days, which presented a risk of death 2.6 times higher, and the PIM 2 score, in which the risk of death increases by 2% for every 1% increase in PIM 2. The risk of death was 83% lower in patients that received EN for 50% of LOS. The program motivated an increase in EN and a decrease in PN use, mainly after implementation of NST and reduced in-PICU mortality rate
**Kiss et al, 2014**<br>U.S.A.,<br>PCICU	Randomised controlled trial	To decrease skin breakdown in the PCICU patient population through the standardization of nursing practice; to create and implement a practice guideline for PCICU nurses and providers; to identify gaps in the literature to guide further evidence-based research towards best practices for paediatric skin care, to decrease the negative outcomes from skin breakdown in the PCICU	n(1)=100 (pre-implementation), admitted PCICU between May 2012 and October 2012; n(2)=100 (post-implementation) admitted between January 2013 and June 2013; aged between 0 and 18 years	Pressure ulcers (Prevalence and/or incidence rate)	Application of a guideline for practice, which guided PICU nurses on the potential interventions for skin-breakdown issues	Patients admitted to the PCICU before implementation of the protocol were 1.35 times more likely to have skin breakdown than those admitted after implementation. The post-implementation patients had a significant decrease in skin breakdown; There was a 41% incidence of skin breakdown before implementation of the protocol and an 18% incidence after implementation. There was a 25% incidence of pressure ulcers before implementation of the protocol and a 4% incidence after implementation
**Curley et al, 2015**<br>Pennsylvania (U.S.),	Randomised clinical trial	To determine whether critically ill children	n=2,449 children (mean age, 4.7 years;	DOV (IMV-days)	Protocolised Sedation	Duration of mechanical ventilation was not different between the groups (intervention 6.5

PICU		managed with a nurse-implemented, goal-directed sedation protocol experience fewer days of mechanical ventilation than patients receiving usual care	range, 2 weeks to 17 years) mechanically ventilated for acute respiratory failure; n=1,225 patients (protocolized sedation); n=1,224 patients (sedation usual care)	Time to recovery from acute respiratory failure (NR)<br>WD-mechanical ventilation (WD-hours)<br>Post-extubation stridor (Prevalence and/or incidence rate)<br>Pressure ulcers (Prevalence and/or incidence rate)<br>Days of opioid administration (Opioid-days)<br>LOS-PICU (days)<br>Hospital LOS (days)<br>Mortality (PRISM3 score)<br>Level of sedation (State Behavioural Scale)<br>Sedation-related adverse events (Number of events)<br>Measures of sedative exposure:<br>Wake fulness (State Behavioural Scale)<br>Pain (FLACC, Individualized Numeric Rating Scale, Wong-Baker Faces Pain Scale)<br>Agitation (State Behavioural Scale)<br>Occurrence of iatrogenic withdrawal (Withdrawal Assessment Tool–Version 1)		days; control 6.5 days). Sedation-related adverse events including inadequate pain and sedation management, clinically significant iatrogenic withdrawal, and unplanned endotracheal tube removal were not significantly different between the 2 groups. Intervention patients experienced more post-extubation stridor (7% vs 4%) and fewer stage 2 or worse immobility-related pressure ulcers (<1% vs 2%). Intervention patients had fewer days of opioid administration (9 days vs 10 days), were exposed to fewer sedative classes and were more often awake and calm while intubated (86% of days vs 75% of days) than control patients; however, intervention patients had more days with any report of a pain score 4 (50% of days vs 23% of days)
**Aljeesh et al., 2014**<br>Palestine,<br>PICU	Prospective study (pre-post)	To develop interventions to decrease HAI and develop effective prevention strategies	n=26 full-time staff working in the paediatric ICU	HAI (Nosocomial infection rates per 1000 ICU-days)	To identify possible causes of HAI, to identify preventive measures that could decrease HAI and foster appropriate and effective use of antibiotics in the ICU; intervention application	Significant decrease in the HAI and antibiotic use after implementing the intervention program; 76 (63%) positive cultures out of the 120 cultures collected during the year before the intervention, with a nosocomial infection rate of 208 per 1,000 ICU days. The infection rate significantly decreased from the first to the second year following the implementation of the intervention (208 vs 120.55). The mean number of antibiotic vials used before the intervention

						was 130 vials per week, after the intervention, the mean number of antibiotics used weekly was 92 vials, representing a significant decrease in antibiotic use
**Siow et al, 2013** Singapore, MICU SICU	Prospective study	To examine the associations between continuity in nursing care (CINC), defined as fewer nurses caring for a patient, and patient outcomes in a paediatric intensive care unit (ICU)	n=332 patients, from March 2004 to December 2012, age between 7 month-11 years	LOS-PICU (days)<br>DOV (MV-days)<br>ICU-acquired infection (CLABSI, VAP, CAUTI) (Prevalence and/or incidence rate)<br>Mortality (PIM2 and PRISM3)	Continuity in Nursing Care (CINC)	The median LOS and the length of ventilator days were the shortest in patients with the 1st quartile of Continuity of Care Index (CCI); Of the total sample, 58% of the patients experienced at least 1 adverse event in the ICU, with an adverse event rate of 1.66 per 100 patient days. In this data set, 11% of patients experienced at least 1 ICU-acquired infection. There was a total of 144 patients with central venous catheters. Of these cases, 17% experienced a CLABSI. Of the 232 subjects who were intubated and supported on mechanical ventilation, 3% experienced VAP. There were 196 subjects who had a bladder catheter inserted. Of these cases, 7% experienced CAUTI. There was a positive association between the occurrence of ICU-acquired infection and CINC, and this association was marginally significant. CINC was associated with a longer ICU stay and longer ventilator days but was significantly associated with fewer nurse-sensitive adverse events; sicker patients were more likely to receive more CINC. Continuity in providers may have the potential to affect patient outcomes. More studies are needed to explore this relationship
**Tume et al, 2011** United Kingdom, PICU	Prospective cohort study	To investigate the effect of five selected intensive care nursing interventions on the intracranial pressure (ICP) of moderate to severe traumatic brain-injured children in intensive care	n=25 children (aged 2–17 years) with moderate to severe traumatic brain injury and intraparenchymal ICP monitoring in intensive care	The change in ICP from baseline to maximal during the procedure and 5 min after the procedure (Measurement in mm/hg)<br>The change in MAP and CPP (Measurement in mm/hg)<br>The time to recovery of baseline ICP values after the procedure (NR)<br>6-month neuropsychological outcome (Glasgow Outcome Score (GOS))	5 interventions: endotracheal suctioning and manual ventilation (ETSMV), turning via a log-rolling(LR)approach, eye care, oral care, washing	ETSMV and LR were associated with clinically and statistically significant changes in ICP from baseline to maximal ICP and from maximal post-ICP. Eye care, oral care or washing did not cause any clinically significant change in ICP from baseline. Only two of the five nursing interventions, endotracheal suctioning and LR, caused intracranial hypertension in moderate to severe traumatic brain-injured children, and after craniectomy, no care interventions cause any significant change in ICP

**Sener et al, 2019** Turkey, PICU	Randomised controlled trial	To determine the effect of oral care using chlorhexidine, vitamin E and honey on oral mucositis (OM) management in children treated in a paediatric intensive care unit (PICU)	n=150 children were randomly divided into six groups based on the oral care solutions used (n = 25 in each group), the median age of the participants was 7.25 years	Oral mucositis (OM) (Classification of mucositis with World Health Organization Oral Mucositis Index)	The use of chlorhexidine, vitamin E and honey on oral mucositis (OM)	The mucositis index values of the children treated with vitamin E were significantly lower than in the other groups, while those of the children given chlorhexidine were significantly higher than in the other groups. Vitamin E was determined to be the most effective agent in OM management, followed by honey as the second most effective agent. Chlorhexidine was found to be less effective in OM management compared to the other two agents
**Keogh et al, 2003** Australia, PICU	Intervention study (pre-post)	To standardise the paediatric intensive care unit team's approach to weaning paediatric patients from mechanical ventilation. The aim of this study was to develop, implement and evaluate collaborative guidelines for weaning children from mechanical ventilation	n=220 patients (pre-intervention n=107 and post-intervention n=113), age between 2 month-16 years	Total ventilation time (TVT) (TVT-hours) Weaning duration (WD) (WD-hours) LOS -PICU (days) Quality indicators: weaning failure (Number of events) and reintubations (Prevalence and/or incidence rate)	Application of collaborative weaning guidelines and weaning algorithm	The outcome indicators TVT and LOS, compared between the pre- and post-intervention groups, were longer post-intervention. WD was comparable between groups. Quality indicators were better post-intervention. Kaplan–Meier survival analysis demonstrated that long-term ventilated patients' post-intervention had a reduced probability of remaining ventilated
**Vavilala et al, 2014** Washington (U.S.)	Retrospective cohort study	To develop a set of acute care guideline–influenced clinical indicators of adherence and tested the relationship between these indicators during the first 72hours after hospital admission and discharge outcomes about severe paediatric traumatic brain injury (TBI)	n=236 Children under 18 years (0–17 years old) with severe traumatic brain injury (TBI) who received tracheal intubation for at least 48 hours in the ICU	Discharge survival (Prevalence and/or incidence rate) Cognitive status (Glasgow Outcome Scale (GOS) score) Hospital mortality (NR)	Paediatric Guidelines	Acute care clinical indicators of adherence to the Paediatric Guidelines were associated with significantly higher discharge survival and improved discharge Glasgow Outcome Scale. Some indicators were protective, regardless of treatment location, suggesting the need for an interdisciplinary approach to the care of children with severe traumatic brain injury

**Cirulis et al, 2016** Utah (U.S.), PICU	Retrospective cohort study	Evaluation of the VAE algorithm in paediatric patients	n=119 Pediatric (ages 0–18) trauma patients with moderate-to-severe traumatic brain injury (TBI) ventilated for ≥2 days	VAP (Prevalence and/or incidence rate)	Surveillance Ventilator-associated-events (VAE)	Both current and modified VAE criteria have poor sensitivity but good specificity in identifying paediatric VAP. Thirty-nine (33%) of 119 patients developed VAP. Sensitivity of the adult ventilator-associated condition (VAC) definition was 23%, which increased to 56%, using the modified paediatric (VAPMP) criterion. Specificity reached 100% for both original and MP probable VAP using VAE criteria. Children who developed VAP or VAC had similar baseline characteristics; Diagnosis of VAP and VAC portended similarly unfavourable outcomes
**Jensen et al, 2018** Denmark, PICU	Randomized controlled trial	To compare two PEWS in a multicentre study	n=16,213 (random sample) paediatric patients (0–19 years)	The primary outcome: The sum of hospitalized children experiencing in-hospital clinical deterioration requiring transfer to a higher level of care (PEWS and CDR PEWS)<br>The secondary outcomes:<br>Mortality (Pediatric index of mortality score 3 (PIM3))<br>Severity of illness during the PICU stay based on invasive ventilation and inotropes (NR)<br>LOS-hospital (days)<br>LOS-PICU (days)<br>Continuous Positive Airway Pressure (CPAP) (Number of events)<br>Extra Corporal Membrane Oxygenation (ECMO) (Number of events)	The Bedside PEWS or Central Demark Region (CDR) PEWS	Of the 21,077 patients who met the inclusion criteria, 16,213 were enrolled, as 4,861 were excluded after randomization: 4,631 were randomized, but their consent was not obtained, and 230 were randomized but were never admitted to the hospital. In the modified intention-to-treat primary outcome analysis, 4910 admissions were included in The Bedside PEWS group and 5,799 in the CDR PEWS group. We identified 719 transfers, but of these only 22 unplanned transfers owing to clinical deterioration were identified. Of the 22 patients 14 patients were in The Bedside PEWS group, and 8 in the CDR PEWS group. Of the 22 patients, 8 and 4 patients in The Bedside PEWS and CDR PEWS groups, respectively, did not have any PEWS score registered. Additionally, 1 patient in The Bedside PEWS group and 2 in the CDR PEWS group only had PEWS scores registered within the final hour before transfer. We found a difference in time from admission to transfer, with a median of 1670 min for The Bedside PEWS group and 412 min for the CDR PEWS group. In the comparison of the two ROC curves, no significant difference in accuracy of predicting unplanned transfer due to clinical deterioration was found. The CDR PEWS prevents as many critical events as The Bedside PEWS. Shorter median time to PEWS reassessment when CDR PEWS was used, and fewer reassessments being done to late could

						reflect that the CDR PEWS was more acceptable to staff
**Steffen et al, 2017** Missouri (U.S.), PICU	Randomised controlled trial	To verify that EPR strategies, facilitated by implementation science methods, would decrease excess blood drawn and reduce transfusion frequency	Preintervention (n =112) Postintervention (n =111), patients <18 years old	Volume overdrawn (Millilitre/kilogram per PICU day)<br>Incidence of RBC transfusion (Prevalence and/or incidence rate)	Excessive phlebotomy reduction (EPR) strategies. The interventions included: (1) the development of a bedside reference guide that included minimal volumes for >50% of the most common laboratory combinations; (2) the use of a closed-loop system to minimize blood discarded from central venous catheter blood draws; (3) the use of microtubes when possible; (4) a standardization of the blood volume for cultures	EPR strategies decreased blood overdraw volumes 62% from 5.5 mL preintervention to 2.1 mL postintervention. Fewer patients received red blood cell transfusions postintervention (32.1% preintervention versus 20.7% postintervention). Regression analyses showed that EPR strategies reduced blood overdraw volume and lowered transfusion frequency. Postintervention surveys reflected a high degree of satisfaction (93%) with EPR strategies, and 97% agreed EPR was a priority postintervention
**Feng et al, 2020** China, PICU	Prospective cohort study	To investigate the diagnostic value of two malnutrition screening tools: STRONGkids (Screening Tool for Risk of Nutritional status and Growth) and STAMP (Screening Tool for the Assessment of Malnutrition in Paediatrics) in assessing the risk of malnutrition in critically ill children	n=160 paediatric oncology patients admitted to the PICU, children from 30 day to 18years of age.	Primary outcome: Malnutrition risk (Prevalence of malnutrition)<br>DOV (IMV-days)<br>Secondary outcomes: LOS-PICU (days)<br>LOS-hospital (days)<br>Mortality (Pediatric critical illness scores (PCIS))<br>Readmission rate to PICU in 48h (Prevalence and/or incidence rate)<br>HAI (Prevalence and/or incidence rate)	STRONGkids (Screening Tool for Risk of Nutritional status and Growth) and STAMP (Screening Tool for the Assessment of Malnutrition in Paediatrics)	The prevalence of malnutrition was 11.3% according to weight-for-age z-score, 16.3% according to height-for-age z-score, 21.3% according to body mass index-for-age z-score, 14.4% according to weight-for height z-score, 34.4% according to mid-upper arm circumference-for-age z-score. Variations of malnutrition prevalence (11.3 to 34.4%) were noted across different anthropometrical parameters. Currently, there is no gold standard for evaluating nutritional status in children
**Keogh et al, 2015** Australia, PICU	Retrospective study (pre–post)	To develop and implement guidelines for sedation and analgesia management in the PICU and, following this, evaluate the impact, and acceptability and feasibility of their use in the clinical setting	n=75 pre-test, n=63 post-test, Patients admitted to the PICU and ventilated for ≥24h, aged more than 1month and not admitted for seizure management or terminal care	Total ventilation time (TVT-hours)<br>Sedation doses (µg/kg/h) and duration (days)<br>LOS-PICU (days)<br>Quality indicators: accidental extubation (Number of events) and readmission rates (Prevalence and/or incidence rate)	Guidelines for sedation and analgesia management for critically ill children including algorithm and assessment tools. Guideline adherence was assessed through chart audit and staff were surveyed about merit and the use of guidelines	Analysis revealed differences in median Morphine infusion duration between groups, pretest 3.63 days (87h) vs post-test 2.83 days (68 h), and maximum doses, pretest 120 µg/kg/h vs post-test 97.5 µg/kg/h. There were also no differences identified between the TVT and LOS for each group. There were no incidents of accidental extubation or readmission within 48 h for participants in either group for the study
**Ramazani et al, 2018** Iran,	Prospective cohort study	To compare the predictive ability of these three models	n=90, the mean age of the cohort was 7.80 ±	Survival and mortality indices (SOFA, GCS, and	SOFA (Sequential Organ Failure Assessment), GCS	The SOFA, GCS and FOUR scores between survivors and non survivors, were statistically

MICU<br>SICU		for predicting medical /surgical ICU mortality in critically ill children	4.43 years (range 2–18years) an ICU length of stay (LOS) less than 24h	FOUR scores in the first 24h of ICU admission)	(Glasgow Coma Scale), FOUR (Full Out line of Un Responsiveness)	different. The discrimination power for SOFA, GCS and FOUR score was moderate. The only well calibrated model was GCS. The performance of the three predictive models SOFA, GCS and FOUR score for predicting outcomes in children admitted to PICUs was good. The discrimination was moderate for all three models, and calibration was good just for GCS. GCS was superior in predicting outcome in critically ill children
**Tume et al, 2017**<br>United Kingdom, PICU	Comparative study	To identify whether routine GRV measurement impacted on energy delivery in mechanically ventilated PICU patients and to identify whether routine GRV measurement impacted on the incidence of complications: vomiting, necrotising enterocolitis (NEC) and ventilator acquired pneumonia (VAP)	n=42 in PICU-GRV and n=45 in PICU NoGRV. The median age was 5.3 months (PICU-GRV) vs 9.7 months (PICU-noGRV); the median weight was 5.4 kg (PICU-GRV) vs 9.8 kg (PICU-noGRV)	Vomiting (Prevalence and/or incidence rate)<br>VAP (VAP per 1000 ventilator-day)<br>Necrotising enterocolitis (Number of events)	The measurement of gastric residual volume (GRV)	The practice of routine gastric residual measurement did not significantly impair energy targets in the first 4 days of PICU admission. The incidence of vomiting in the first 4 days was higher in the PICU-GRV but was not statistically significant; VAP rates were similar, 7.01 per 1000 ventilator days (2/42) in PICU-GRVand 5.31 per 1000 ventilator days (3/45) in PICU-noGRV significantly longer length of ventilation in PICU-noGRV However, not measuring GRV did not increase vomiting, ventilator-acquired pneumonia or necrotising enterocolitis, which is the main reason clinicians cite for measuring GRV
**Irving et al, 2018**<br>Pennsylvania (U.S.), PICU	Observational study	The impact of nutrition status on outcomes in paediatric severe sepsis is unclear. We studied the association of nutrition status (expressed as body mass index z score) with outcomes in paediatric severe sepsis	n=417, children less than 18 years with severe sepsis enrolled in the Sepsis Prevalence, Outcomes, and Therapies study	Mortality (90-day mortality)<br>LOS-PICU (PICU LOS-90 days)<br>New or progressive multiple organ dysfunction syndrome (NPMODS) (Prevalence and/or incidence rate)<br>Vasoactive-free days (VAFDs) (Prevalence and/or incidence rate)<br>Ventilator-free days (VFDs) (Prevalence and/or incidence rate)	NR	Of the 567 patients with severe sepsis enrolled in the SPROUT study, complete nutrition status data were available for 417 patients. There were no significant differences in patient characteristics, ICU therapies, and outcomes between patients with complete nutrition status data. The proportions of patients with undernutrition, normal nutrition, and overnutrition were 30%, 33%, and 37%, respectively. In patients with complete nutrition status data, ICU mortality was 25% (103 deaths), whereas the median ICU LOS in survivors (n = 314) was 14 days. Severe undernutrition was independently associated with higher all cause ICU mortality in children with severe sepsis. In contrast, severe overnutrition was independently associated with longer ICU LOS in survivors of paediatric severe sepsis
**Agus et al, 2017**<br>Philadelphia (U.S.), PICU	Randomised controlled trial	To show the effects of a tight glycaemic control in a critically children	n=713 patients, n1=360 lower-target group (80-110) and n2=353	The primary outcome:	Tight glycaemic control, the blood glucose level was controlled with the use of	Critically ill children with hyperglycaemia did not benefit from tight glycaemic control targeted to a blood glucose level of 80 to 110 mg per

			higher-target group (150-180), from 2 weeks to 17 years	The number of intensive care unit (ICU)–free days to day 28.<br>Secondary outcomes:<br>Mortality (90-day mortality)<br>Severity of organ dysfunction<br>The number of ventilator-free days to day 28<br>The incidence of HAI to day 28<br>Incidence of hypoglycaemia (Prevalence and/or incidence rate)	continuous intravenous regular human insulin	decilitre, as compared with a level of 150 to 180 mg per decilitre. No significant differences were observed in mortality, severity of organ dysfunction, or the number of ventilator-free days. The median number of ICU-free days did not differ significantly between the lower-target group and the higher-target group
**Sulla et al, 2018** Italy, PICU	Comparative study	To assess the applicability of the BPS for use with paediatric patients	n=9 patients, 5 boys and 4 girls, all in their first year of age; 39 observations were collected; non-verbal, sedated and mechanically ventilated critical care paediatric patients	Pain control and assessment in the paediatric patient (Critical-Care Pain Observation Tool and/or Numeric Rating Scale)	A questionnaire was administered to physicians and nursing staff that work in the units where the study was conducted in order to investigate the actual use of observational pain scales in their units. A second questionnaire was administered to a group of experts regarding the BPS, to assess both face validity and content validity, and to gain opinions on the relative appropriateness of each item	Only 46% of respondents stated that they assessed patients' pain levels, with an average of 2.8 times per shift; 60% of respondents declared to be unhappy with the observational scales that they utilise. Correlations between BPS and the other instruments were high, demonstrating a good concurrent validity of the test. Although the current study is based on a small sample of participants, these first results encourage us to continue working in the validation of the BPS in paediatric patients
**Phipps et al, 2005 West Virginia** (U.S.) PICU	Randomised controlled trial	To compare 3 different techniques used to place nasojejunal (NJ) feeding tubes in the critically ill or injured paediatric patients	n=75 paediatric patients, studied from March 2001 until March 2002. n=25 standard technique facilitated with the use of pre-insertion erythromycin; n=24 standard technique facilitated with gastric insufflation; n=26 standard technique. Middle age: 47 months	The primary outcome: the successful placement of the nasojejunal (NJ) tube on the first or second attempt (Number of events)	3 different techniques used to place nasjejunal (NJ) feeding tubes in the critically ill or injured paediatric patients: standard technique facilitated with gastric insufflation, and standard technique facilitated with the use of pre-insertion erythromycin	94.6% (71/75) of tubes were successfully passed into the small bowel on the first or second attempt. Evaluation of the data revealed no significant association with a specific technique and successful placement; the majority of NJ feeding tubes can be placed in critically ill or injured children on the first or second attempt, regardless of the technique used
**Tume et al, 2010** United Kingdom, PICU	Prospective observational study	To investigate (a) how actual calorie intake compared with estimated caloric	n=47 paediatric patients, middle age 10 months, that was	Reduced energy intake (Prevalence and/or incidence rate)	Enteral Nutrition	Only 47% of the children had enteral feeds started within our 6 h post-admission target. Over half (55%) of the children received less

		requirements and (b) whether feeding guideline adherence resulted in improved nutritional intake	admitted to PICU and who stayed for more than 24 h			than half of their estimated calorie requirements. This study found that many children are not receiving adequate nutrition in PICU and that the use of feeding guidelines significantly improves calorie delivery in PICU patients
**Brown et al, 2018** U.S., PICU	Prospective, randomised, comparative study	To compare the effectiveness and safety of C-GF vs B-GF in intubated paediatric patients	n=25 patients (B-GF =11; C-GF =14), randomized aged 1 month–12 years who were intubated within 24 hours and received EN starting within 48 hours of admission to a C-GF or B-GF group	Aspiration events (Number of events) Lung injury (Number of events) Morbidity (NR)	Bolus gastric feeding (BGF), continuous gastric feeding (C-GF)	At 24 hours, B-GF was associated with higher energy and protein delivery and was associated with faster time to goal volume; No aspiration events resulting in additional lung injury were noted for either group; B-GF was associated with superior delivery of EN with a comparable safety profile to C-GF
**Edwards et al, 2015** New York (U.S.), PICU	Prospective cohort study	To demonstrate the trend of PICU CLABSI rates and reports the IP&C and CL bundle policies and practices of these PICUs and how they related to infection rates	n=88 NHSN hospitals with 99 PICUs from 34 states	CLABSI (CLABSI per 1000 line-days)	The adoption of central line (CL) bundle policies, bundle practices	The CLABSI rates decreased each year from 2006; full compliance with CL prevention practices is associated with lower CLABSI rates compared with less than full compliance. There was a nonsignificant trend in decreasing CLABSI rates as PICUs improved bundle policy compliance. Given that few PICUs reported full compliance with these policies, PICUs increasing their efforts to comply with these policies may help reduce CLABSI rate
**Düzkaya et al, 2016** Turkey, PICU	Randomised controlled trial	To compare the effectiveness of chlorhexidine-impregnated dressings with that of standard dressings in preventing catheter-related bloodstream infections	n=100 children were randomly divided into 2 groups of 50 each: a chlorhexidine group and a standard group; 63% were 1 month to 1 year old.	Primary outcomes: Catheter colonization (Prevalence and/or incidence rate) CRBSIs (Number of events) Secondary outcomes: Durations of IMV (DOV) (IMV-days) LOS-PICU (days) Duration of catheterization (Catheterization-days)	The use of chlorhexidine-impregnated dressings and the use of standard dressing	Catheter colonization occurred in 4 patients in the standard group (8%) and in 1 patient in the chlorhexidine group (2%). Catheter-related bloodstream infections occurred in 5 patients in the standard group (10%) and in 1 patient in the chlorhexidine group (2%). Use of chlorhexidine-impregnated dressings reduced rates of catheter-related bloodstream infections, contamination, colonization, and local catheter infection in a PICU and had a shorter ICU length of stay, shorter duration of mechanical ventilation, and shorter duration of catheterization, but was not significantly better than use of standard dressings
**Traube et al, 2014** New York (U.S.), PICU	Randomised controlled trial	To determine validity and reliability of the Cornell Assessment of Paediatric Delirium, a rapid observational screening tool	n=111 patients stratified over ages ranging from 0 to 21 years and across developmental levels	Prevalence and screening of delirium (The Cornell Assessment of Paediatric Delirium)	248 paired assessments completed; Double-blinded assessments were performed by nursing staff in the PICU. These ratings were compared with an assessment by	The Cornell Assessment of Paediatric Delirium had an overall sensitivity of 94.1% and specificity of 79.2%; The Cornell Assessment of Paediatric Delirium takes less than 2 minutes to complete. Prevalence of delirium by psychiatric assessment was 20.6% (n = 51). The Cornell Assessment of Paediatric Delirium is a valid,

					consultation liaison child psychiatrist using the Diagnostic and Statistical Manual IV criteria as the "gold standard" for diagnosis of delirium	rapid, observational nursing screen that is urgently needed for the detection of delirium in PICU settings
**Manning et al, 2015** Massachusetts (U.S.), PICU	Retrospective cohort study	To identify factors associated with development of occipital pressure ulcers in acutely ill infants and children	n=60 patients, the median age of the sample was 12 months, most of the patients were less than 1 year old; were critically ill, requiring high-risk therapies and had multiple medical devices in place; Among the patients, 86% were in the intensive care unit with cardiovascular or pulmonary problems	Risk for occipital pressure ulcers (Cases of occipital pressure ulcers during a 4-year period)	Charts of all patients with occipital pressure ulcers reported in a computerized safety event reporting system since its implementation in 2005 and of any patients with such ulcers recalled by members of the skin care special interest group were reviewed retrospectively	During a 4-year period, 60 cases of occipital pressure ulcers were identified: 40% stage I, 12% stage II, 30% unstageable, and 18% deep tissue Injury; A total of 68% had comorbid conditions. Patients with the ulcers were commonly treated with mechanical ventilation (83%) and sedation (74%) and were described as agitated (42%). Many of these patients were receiving vasoactive medications (50%) and had vascular access devices in the neck that restricted head movement (45%). Infants and children at risk for occipital pressure ulcers can be prospectively identified, allowing implementation of nursing interventions to prevent these ulcers
**Colwell et al, 2018** California (U.S.), PICU	Prospective observational study	To implement a standardized mobilization therapy protocol in a paediatric intensive care unit and improve mobilization of patients	n=567 patient encounters were analysed, in 9 months, 294 (52%) of which achieved goal mobilization	Mobilization Protocol adherence (Number of events)<br>Adverse events (Number of events)<br>Barriers to mobilization (Non reported)	Unique goal-directed multidisciplinary and multiprofessional mobilization protocol for critically ill infants and children admitted to the PICU	A multidisciplinary, multiprofessional, goal directed mobilization protocol achieved goal mobilization in more than 50% of patients in this paediatric intensive care unit. In 9 months, 567 patient encounters were analysed, 294 (52%) of which achieved goal mobilization. The mean ratio of mobilization level to severity of illness improved slightly but non significantly. Encounters that met mobilization goals were in younger and more ill patients and were less likely to have barriers than encounters not meeting the goals
**Neunhoeffer et al, 2015** Germany, MICU SICU PCICU	Prospective study (pre-post)	To evaluate the effects of a nurse-driven goal-directed analgesia and sedation protocol for mechanically ventilated paediatric patients (pASP) on duration of mechanical ventilation, paediatric intensive care unit (PICU) length of stay, total doses of opioids and benzodiazepines, and occurrence of withdrawal symptoms	n=337 medical paediatric patients requiring mechanical ventilation with PICU length of stay for at least 24 h were included.	DOV (IMV-days)<br>LOS-PICU (days)<br>Total doses of opioids and benzodiazepines (mg x kg(-1) x day(-1))<br>Occurrence of withdrawal symptoms (Prevalence and/or incidence rate)	Nurse-driven goal-directed analgesia and sedation protocol for mechanically ventilated paediatric patients: nurses managed analgesia and sedation following a PASP, including COMFORT 'behavioural' Scale, Nurse Interpretation Sedation Scale, and Sophia Observation Withdrawal Symptoms Scale	Implementation of a nurse-driven pASP reduced the total dose of benzodiazepines and the occurrence of withdrawal symptoms significantly. Median duration of mechanical ventilation was 2.02 days in the group preceding protocol implementation and 1.71 days afterwards.<br>Median PICU length of stay was 5.8 days in the pre-implementation and 5.0 days in the postimplementation group. Incidence of withdrawal was significantly lower over the postimplementation period

**Kristen et al, 2018** New Jersey (U.S.), PCICU	Narrative review	To increase awareness of delirium in the PCICU and give nurses the tools to identify it and intervene when necessary. Available screening tools and environmental and pharmacological interventions are explored	NR	Delirium (Confusion Assessment Method for the Intensive Care Unit (ps/pCAM-ICU) and Cornell Assessment of Pediatric Delirium (CAPD))<br>LOS-Hospital (days)<br>Morbidity (NR)<br>Mortality (NR)	"BRAIN MAPS" is an acronym designed to managing delirium	The ps/pCAM-ICU is a more rapid assessment and thus can be used at any point of concern for delirium, while the CAPD requires observation of the patient over approximately 4 to 6 hours. After screening, nurses can use the BRAIN MAPS acronym to evaluate the possible cause of delirium in order to intervene appropriately. BRAIN stands for Bring oxygen, Remove/Reduce delirium-causing drugs, Atmosphere, Infection/Immobilization/Inflammation, and new organ dysfunction. MAPS stands for Metabolic disturbances, Awake, Pain, and Sedation. Environmental interventions can also be used as preventive measures
**Malyon et al, 2014** Australia, PICU	Prospective cohort study	To address a gap in the literature about current practice in the securement and dressing of PIVCs in paediatric acute care, and to ascertain the duration and failure of these devices	n=458 patients aged 0–15 years, who had a PIVC inserted prior to emergent admission to the hospital	The primary outcomes were:<br>(1) PIVC dwell time (PIVC-h)<br>(2) PIVC failure (occlusion, infiltration, phlebitis, dislodgement or infection) (Prevalence and/or incidence rate)<br>(3) dressing and securements used (Prevalence and/or incidence rate)	Peripheral intravenous catheters (PIVCs)	Of 458 participants, median device duration was 29 h; 113/456 (24.8%) of PIVCs were removed due to device failure, presenting as: infiltration (65/456, 14.3%); accidental dislodgement (23/456, 5.0%); blockage (12/456, 2.6%); phlebitis (7/456, 1.5%); or 'other' (6/456, 1.3%). PIVC placement in the antecubital fossa, in comparison to the hand, was significantly associated with an increased risk for failure. PIVC securement and dressings were predominantly bordered polyurethane dressings and splints (457/458, 99.8%)
**Curley et al, 2003** Massachusetts (U.S.), PICU	Prospective cohort study	(a) To establish the predictive validity of the Braden Q Scale in an acutely ill paediatric population<br>(b) To determine the critical cut-off point for classifying patient risk<br>(c) To determine the best time to assess patient risk	n=322 patients on bedrest for at least 24 hours without pre-existing pressure ulcers or congenital heart disease	Pressure ulcers (Prevalence and/or incidence)	The Braden Q Scale, patients were observed up to 3 times per week for 2 weeks and then once a week until PICU discharge for a median of 2 observations reflecting 887 skin assessments	n=87 (27%) developed 199 pressure ulcers; n= 139 (70%) were Stage I pressure ulcers, n= 54 (27%) were Stage II, and n= 6 (3%) were Stage III. Most pressure ulcers (57%) were present at the first observation. The performance of the Braden Q Scale in a paediatric population is similar to that consistently reported for the Braden Scale in adult patients. The Modified Braden Q Scale, with 3 subscales, provides a shorter yet comparable tool
**Snyder et al, 2020** Pennsylvania (U.S.), PICU	Prospective observational study	To increase bundle compliance by using targeted rounds specifically focused on eliminating dependent loops in drainage tubing and ensuring appropriate catheter use to reduce the incidence of	A multidisciplinary team was formed to identify misperceptions, highlight best practices, and eliminate barriers to success over 1 year in	CAUTI (CAUTI per 1000 catheter-days at baseline to 0)	CAUTI bundle	Bundle compliance supported by targeted rounding increased from 84% to 93% and helped reduce the overall catheter-associated urinary tract infection rate from 2.7 infections per 1,000 catheter-days at baseline to 0 catheter-days. Targeted rounding for paediatric patients with an indwelling urinary catheter is an effective

		catheter-associated urinary tract infections	a single PICU. The PICU CAUTI working group consisted of an attending physician, nurse practitioner, unit-based clinical nurse specialist, unit-based safety quality specialist, clinical nurse leader, staff nurse, infection control specialist, executive sponsor, and data analyst			and sustainable strategy to reduce catheter-associated urinary tract infections
**Ge et al, 2017** China, PICU	Systematic review	To evaluate the psychometric properties of subjective sedation scales using one psychometric scoring system to identify the appropriate scale that is most suitable for clinical care practice	Articles that had been published or were in press and discussed the psychometric properties of sedation scales were included. The population comprised critically ill infants and non-verbal children ranging in age from 0 to 18 years who underwent sedation in a PICU	Depth of sedation (Comfort Scale and Comfort Behaviour Scale)	NR	43 articles were included in this review and 13 sedation scales were examined. The quality of the psychometric evidence for the Comfort Scale and Comfort Behaviour Scale was 'very good'; the Comfort Scale and Comfort Behaviour Scale are useful tools for measuring sedation in paediatric patients
**Kusahara et al, 2014** Brazil, PICU	Prospective cohort study	To identify predisposing factors for ventilator associated pneumonia in children	n=96 patients, age 1 month- 16 years	VAP (Clinical Pulmonary Infection Score (CPIS) and Centres for Disease Control and Prevention and the National Healthcare Safety Network (CDC/NHSN))	Variables examined were demographic characteristics, inpatient care, medications, nutrition, invasive procedures, and characteristics of mechanical ventilation. Data were analysed by using Pearson $\chi 2$ analysis, Fisher exact and Mann-Whitney tests, odds ratios, and forward stepwise logistic regression	n = 65 patients without VAP, n = 31 patients with VAP. Use of vasoactive drugs, presence of a naso-enteral tube, and duration of stay in the paediatric intensive care unit were independent risk factors for ventilator-associated pneumonia
**Düzkaya et al, 2017** Turkey, PICU	Intervention study (pre-post)	To review the effect of standardized oral care, which was implemented in line with the oral health care guide in children in intensive care to prevent mucositis formation	n=284 patients post-intervention; n=310 patient Pre-intervention; age 1 month - 17 years	Oral mucositis (OM) (Prevalence and/or incidence rate)	Daily oral care with an oral care guide developed by the researchers, oral care guide (OCG)	OM occurred in 16 (5.2%) patients in the pre-intervention group and 7 (2.5%) in the post-intervention group, 10 patients had grade 1, and 6 patients had grade 2 oral mucositis in the pre-intervention group, and in the post-intervention group, 3 patients had grade 1, and 4 patients had grade 2 oral mucositis. Oral mucositis can be reduced through the practice of administering

						oral care in accordance with oral healthcare guidelines
**Ullman et al, 2011** Australia, PICU	Prospective cohort study	To describe the oral health status of critically ill children over time spent in the paediatric intensive care unit, examine influences on the development of poor oral health and explore the relationship between dysfunctional oral health and healthcare-associated infections	n=46 patients, aged between 11 month-14 years	Oral health status (Oral Assessment Scale and culturing oropharyngeal flora)<br>PICU-related HAI (Prevalence and/or incidence rate)<br>Oral colonisation by pathogenic organisms	The oral health of the study participants was described using the Oral Assessment Scale (OAS) and second daily culturing of oropharyngeal flora within 12 hours of admission to PICU and then every second day for the course of their admission to PICU; Critical illness severity was described using the Paediatric Logistic Organ Dysfunction score (PELOD) and the Paediatric Indicator of Mortality 2 (PIM2)	Of the 46 participants, 63% (n=32) had oral dysfunction and 41% (n=19) demonstrated pathogenic oropharyngeal colonisation during their critical illness. The severity of critical illness had a significant positive relationship with pathogenic and absent colonisation of the oropharynx. 63% of healthcare-associated infections involved the preceding or simultaneous colonisation of the oropharynx by the causative pathogen
**Brown et al, 2017** Ohio (U.S.), PICU	Intervention study (pre-post)	To determine the effectiveness of the electromagnetic device in reducing the numbers of radiation exposures, intrahospital transports, tubes used, and tube placement attempts in the paediatric intensive care unit	n=73 children (n=30 control group, n=43 intervention group)<br>Patients < 18 years of age and weighing at least 3 kg who required a post-pyloric feeding tube	Primary outcome:<br>Number of radiation exposures (Number of events)<br>Secondary variables:<br>LOS-PICU (days)<br>Success of PPFT placement (Prevalence and/or incidence rate)<br>Number of attempts required for successful placement (Number of events)	Independent-samples Student t tests were used to compare the 2 study groups for mean equality	The intervention group had a higher success rate at tube placement and fewer radiation exposures, intrahospital transports, and tubes used. The percentage of successful insertion attempts out of the total attempts was significantly higher in the intervention group; Use of the electromagnetic device was also cost-effective. No pulmonary or other placement-related adverse events were reported
**Wieczorek et al, 2016** Maryland (U.S.), PICU	Intervention study (pre-post)	To determine the safety and feasibility of an early mobilization program in a paediatric intensive care unit (PICU)	n=200 children aged 1 day through 17 years and had a length of stay in PICU of at least 3 days. n=100 pre-implementation group (675 PICU days) and n=100 post-implementation group (737 PICU days)	The primary outcome measures were<br>1) the proportion of patients with OT and/or PT consultations by PICU Day 3<br>2) the number and types of mobilization activities performed by PICU Day 3.<br><br>Secondary outcome measures were<br>1) the number of times and reasons that activities were stopped	A multicomponent, interdisciplinary, and tiered activity plan to promote early mobilization of critically ill children. Activities were categorized as in-bed therapies or mobility therapies. In-bed therapies included passive range of motion, active range of motion, and active or passive bed positioning, and splinting. Mobility activities included sitting at edge of bed, sit to stand,	The implementation led to an increase in occupational therapy consultations (44% vs 59%) and physical therapy consultations (54% vs. 66%) by PICU day 3. More children engaged in mobilization activities after the "PICU Up!" intervention by PICU day 3, including active bed positioning and ambulation. No adverse events occurred as a result of early mobilization activities. The most commonly reported barriers were availability of appropriate equipment. The program was positively received by PICU staff

				2) barriers to mobilization activities<br>3) mobilization-related adverse events, including inadvertent extubation or line removal (number of events)	transfer, ambulation, and play	
**Dokken et al, 2013**<br>Norway,<br>PICU	Prospective, observational study	To examine the prevalence of underfeeding, adequate feeding, and overfeeding in mechanically ventilated children and to identify barriers to the delivery of nutrition support	n=30 mechanically ventilated children, aged 3 month – 14 years who fulfilled the criteria for indirect calorimetry	Underfeeding, defined as <90% of required kcal/d (Prevalence and/or incidence rate)<br>Adequate feeding as 90%–110% of required kcal/d (Prevalence and/or incidence rate)<br>Overfeeding as >110% of required kcal/d (Prevalence and/or incidence rate)	104 calorimetric measurements for 140 total days were recorded for 30 mechanically ventilated children. Actual energy intake was recorded and compared with the required energy intake. The reasons for interruptions to enteral and parenteral nutrition were recorded	Underfeeding, adequate feeding, and overfeeding occurred on 21.2%, 18.3%, and 60.5% of the 104 measurement days, respectively. Fasting for procedures was the most frequent barrier that led to interrupted nutrition support
**Chuengchitraks et al, 2010**<br>Thailand,<br>PICU	Prospective cohort study	To demonstrate the incidence of catheter-related blood stream infection (CRBSI) of patients in the paediatric intensive care unit (PICU) after implementing the new guideline to prevent CRBSI	n=61 children who had central venous catheter (CVC) inserted from the operation room before admission or CVC placed in the PICU	CRIBSI (Rate of CRBSI for 1,000 catheter days)<br>Hematoma (Prevalence and/or incidence rate)	The guideline included five key components (hand hygiene, maximal barrier precautions, povidone skin antiseptic, optimal catheter site selection, daily review of line necessity with prompt removal of unnecessary lines) called "central line bundle"	Average duration of catheterization was 8.7 days. Complications were found in 8 cases (13.1%). Hematoma was the most common complication (6.6%) followed by infection (3.3%). Rate of CRBSI was reduced from 2.6 per 1,000 catheter days to 2.4 per 1,000 catheter days after implementing the new practice guideline. The rate of CRBSI was reduced after implementing the new "central line bundle" guideline to prevent CRBSI
**Fagioli et al, 2018**<br>Italy,<br>PICU	Prospective cohort study	To translate and validate the Italian version of the COMFORT behaviour scale (CBS) in a PICU in terms of its psychometric, construct, feasibility and reproducibility properties	n=35 sedated and mechanically ventilated patients, aged between one month and 17 years	Pain (Comfort Behaviour Scale (CBS))<br>Distress (Nurse Interpretation of Sedation Score (NISS))	Before and after tracheal suctioning, 71 observations were performed on 35 sedated and mechanically ventilated patients; Pain and distress were assessed using the Comfort Behaviour Scale (CBS) and the Nurse Interpretation of Sedation Score (NISS)	Patients were considered adequately sedated with the NISS in 59 observations before tracheal suctioning and in 42 observations after the procedure. Using the CBS as a comparison, patients were considered adequately sedated in 22% (n=13/59) of observations before the procedure and 38% (n=16/42) of observations after the procedure. The Italian version of the CBS proved to be valid and reproducible for the objective measurement of pain and distress in a wide age range of patients admitted to PICUs
**Taha et al, 2011**<br>California (U.S.),<br>PICU	Retrospective descriptive study	To examine the timing of nutritional supplement initiation and the timing of achieving full caloric intake in relation to length of stay	n=109 patients between 8 and 18 years of age with any combination of traumatic injuries	LOS-PICU (days)<br>The disposition status on discharge: mild, moderate, or severe	Nutrition support in children with traumatic injuries	Median time to initiation of nutrition was 1.5 days, and full caloric goals were achieved in 3.4 days. Median ICU LOS was 2.1 days. 48% of patients were discharged home; 28% experienced mild, moderate, or severe disability;

		(LOS) in the intensive care unit (ICU) and patient disposition status at discharge from hospital		disability (Prevalence and/or incidence rate)<br>Mortality (Prevalence and/or incidence rate)		and 24% either died or survived in a vegetative state. Early initiation and achieving full caloric intake were both positively correlated with shorter LOS in the ICU and better disposition status at discharge from the hospital
**Wang et al, 2019**<br>China,<br>PICU	Retrospective cohort study	To investigate the prevalence of medical adhesive-related skin injuries (MARSIs) and associated risk factors in a paediatric ICU (PICU)	n=232 patients, median age 1.07 years, n= 152 (<2 years), n=80 (>2 years)	MARSIs (MARSIs per 1,000 product-days)	Researchers assessed all patients daily for 2 weeks. The use of adhesives and prevalence of MARSIs were recorded. The patients' clinical data were also collected. The prevalence of MARSIs was calculated daily, and the risk factors were examined statistically. 611 person-days were analysed	Prevalence by product ranged from 19 to 53 per 1,000 product-days with a mean of 34 MARSIs per 1,000 product-days. A total of 322 MARSIs were recorded in 227 subjects. The major MARSI types were epidermal stripping and skin tear. The face was the most common MARSI site, and tracheal intubation was the most common inciting condition. Implicated products were acrylate tapes with elastic cloth backings. The children with independent risk factors were females, age 2 years or younger, longer hospital stays, oedema, infection, or surgery
**Vlasselaers et al, 2009**<br>Belgium,<br>PICU	Randomised controlled trial	To investigate the effect of targeting age-adjusted normoglycaemia with insulin infusion in critically ill infants and children on outcome	n=700 critically ill patients (0-16 years); n1=317 infants (aged <1 year) and n2=383 children (aged ≥1 year)	PICU-LOS (days)<br>Inflammation (Prevalence and/or incidence rate)<br>Hypoglycaemia (Number of events)	Patients were randomly assigned by blinded envelopes to target blood glucose concentrations of 2·8–4·4 mmol/L in infants and 3·9–5·6 mmol/L in children with insulin infusion throughout PICU stay (n=349 intensive group), or to insulin infusion only to prevent blood glucose from exceeding 11·9 mmol/L (n=351 conventional group)	Mean blood glucose concentrations were lower in the intensive group than in the conventional group.<br>Duration of PICU stay was shortest in the intensively treated group.<br>The inflammatory response was attenuated at day-5 in the intensive group compared with baseline. The number of patients with extended stay in PICU was 132 (38%) in the intensive group versus 165 (47%) in the conventional group. Hypoglycaemia (defined as blood glucose ≤2·2 mmol/L) occurred in 87 (25%) patients in the intensive group versus 5 (1%) patients in the conventional group; 9 patients (3%) died in the intensively treated group versus 20 (6%) in the conventional group
**Turner et al, 2020**<br>Massachusetts (U.S.),<br>PICU	Retrospective cohort study	To describe PP tube placement and EN practices in a multidisciplinary paediatric intensive care unit (PICU) after the implementation of a nurse-led bedside PP tube-placement program	n=100 patients, the median age was 3.89 years (21 years of age and younger)	Number of encounters for PP tube placement (Number of events)<br>Number of radiation exposures to confirm successful PP tube placement (Number of events)<br>Clinical complications of PP tube placement (oropharyngeal or GI bleeding, bowel injury	Nurse-led bedside PP tube-placement program	Bedside placement was the initial technique for PP tube placement in 93% of patients (successful for 84.9%) and was not associated with serious complications; 87 patients with a PP tube started EN and received a median 73.9% of prescribed energy goal on day 3 after EN initiation. PP EN allowed 14 of 39 patients receiving PN to transition off PN 7 days after EN initiation. Bed side PP tube placement is safe and feasible and allows for effective EN delivery and decreased PN use when applicable

				or perforation, or inadvertent tube placement into the lung) (Prevalence and/or incidence rate)		
**Damme et al, 2018** Michigan (U.S.), PICU	Narrative review	To establish an initial guideline with a set of standards to increase thoughtfulness about mobility in an effort to improve the amount of patient activity, while maintaining a safe environment	The majority of patients were between the age of 1 to 23 months and the primary reason for admission is respiratory dysfunction	Pressure ulcers (Prevalence and/or incidence rate)<br>Infection<br>Loss of muscle mass<br>Critical illness myopathy<br>Overall deconditioning<br>Increase ventilator days<br>LOS-PICU (days)<br>Delirium (Cornell Assessment of Paediatric Delirium (CAPD) tool)	Mobility guideline to developed for use in the paediatric intensive care unit (PICU)	Respiratory dysfunction was the limiting factor for paediatric mobility identified for the PICU patients, has been introduced the assessment of oxygenation index or oxygenation saturation index to determine severity, which was used to align the appropriate phase of mobility. The RASS goal has been placed in the mobility guideline to help identify the appropriate level of activity for the required level of sedation; As sedation and agitation are measured, a patient should also be evaluated for delirium and altered mental status. To safely mobilize, the patient must be cooperative and able to perform a developmentally appropriate activity; to assess delirium with the Cornell Assessment of Paediatric Delirium (CAPD) tool prior to mobility. Contraindications to mobility within the guideline include an open abdomen, unstable fracture or unstable intracranial pressure
**Johnstone et al, 2010** New Zealand, PICU	Narrative review	To improve standards of oral care for children in the PICU. To accomplish this goal, an evidence-based practice process informed by the 1998 Iowa Model was implemented	NR	Increase in dental plaque accumulation (not reported)<br>Bacterial colonization of the oropharynx (not reported)<br>HAI (Prevalence and/or incidence rate)<br>VAP (Prevalence and/or incidence rate)	A survey of nurses was conducted to establish baseline knowledge of oral hygiene and current oral hygiene practices in the PICU. Having identified the problem – poor oral hygiene care in the PICU – a literature search was undertaken to gather relevant literature and research studies.	Across the “best available evidence,” three nursing interventions were identified for oral hygiene care in the paediatric critical care setting: 1) oral assessment, 2) mechanical interventions, and 3) pharmacological interventions. Synthesis of the above literature facilitated the development of an oral hygiene guideline for children in the PICU. Standardized oral hygiene practice has the potential to contribute to improved oral and general health of infants and children in the paediatric critical care setting
**Chamblee et al, 2018** Texas (U.S.), PICU	Narrative review	To provide guidance on using the Braden QD Scale to assess paediatric patients and score their risk of pressure-related injury in numerous scenarios frequently encountered in acute care practice	Paediatric patients in an acute care setting ranging in age from preterm to 21 years	Pressure ulcers (Braden QD Scale (risk identification)	NR	The Braden QD Scale promotes patient safety, quality of care and care monitoring, and effective resource use in paediatric hospitalized patients. The frequency of performing a risk assessment using the Braden QD Scale should be matched to the patient population, following institutional standards; patients who are on bed rest, have limited mobility or sensation, or are critically ill should be assessed at least once

						daily. The Braden QD Scale is an important strategy for ensuring patient safety during hospitalization and presents an opportunity to measure a nurse-sensitive, patient-centred outcome
**Cooper et al, 2013** Maryland (U.S.), PICU	Narrative review	To present the definition, significance, aetiology, risk factors, and diagnosis of VAP in infants and children; to provide background evidence to support the use of intervention and prevention strategies and to explain the rationale for use of these strategies in children; to supply recommendations for a bundled prevention protocol for support of children treated with mechanical ventilation in the PICU, with a focus on suctioning or airway clearance, oral care, and ventilator circuit changes	NR	VAP (Rate of VAP per 1000 intubated patients; rate of VAP per 1000 ventilator days)	NR	The estimated rate of VAP is 1.4 to 5.8 events per 1000 intubated patients, with a downward trend since 2000. In the PICU, 20% of nosocomial infections are VAP, with an incidence of 4 to 44 per 1000 intubated children. Data from the CDC National Nosocomial Infections Surveillance System has indicated a mean PICU VAP rate of 2.9 per 1000 ventilator days. Intubated patients are at risk for VAP because of their poor cough and gag reflexes and their immobility. VAP is associated with increased mortality and morbidity, increased length of hospital-stay, and high health care costs. Risk factors for VAP in children currently include use of opiates for sedation, sustained neuromuscular blockade, use of enteral nutrition, previous antibiotic therapy, the technique used for endotracheal suctioning, reintubation, ventilator circuit changes, gastroesophageal reflux, subglottal or tracheal stenosis, young infants or age greater than 10 years, and trauma or surgical problems. Diagnosis of VAP is based on sputum samples, findings on chest radiographs, and the presence of fever. Interventions: oral hygiene and to remove dental plaque, endotracheal suctioning should be used only when indicated by a physical examination, elevating the head of the bed 30º to 45º to prevent aspiration and minimal changes of the ventilator circuit
**Kawai et al, 2019** Michigan (U.S.), PICU	Randomised controlled trial	To assess the degree of PICU noise pollution, to develop a delirium bundle targeted at reducing noise, and to assess the effect of the bundle on nocturnal noise pollution	n=116 patients, n=8 pilot patients per 40 ICU-days; n=108 non-pilot patients per 28 ICU days; median age 7.0 years	Noise Pollution (Hourly dB readings); Delirium (not reported)	Paediatric delirium bundle; Thirty-five sound sensors were installed in patient bed spaces, hallways, and common areas	Hourly minimum, average, and maximum dB of all occupied bed spaces demonstrated medians of 48.0, 52.8 and 67.0 dB, respectively. Bed spaces were louder during the day than at night. Pilot patient rooms were significantly quieter than non-pilot patient rooms at night. The pilot rooms compliant with the bundle had the lowest hourly night-time average dB. Utilizing the paediatric delirium bundle led to a significant noise reduction that can be perceived as half the loudness with hourly night-time average dB

						meeting the EPA standards when compliant with the bundle
**Rice et al, 2005** U.S., PICU	Narrative review	To offer an overview of the quality and safety of the main PICU treatments	NR	Mortality (PRISM score)<br>LOS-PICU (days)<br>CRBSI (Risk identification)<br>Unplanned extubations (Unplanned extubations rate for 100 ventilator days)<br>Medication safety (NR)	NR	1) The PRISM is used to predict the probability of mortality based on certain physiologic variables. The probability of mortality calculated from the PRISM score does help to describe how acutely ill a child is, and discriminates well whether a child is at high risk of dying;<br>2) The severity of illness as determined by a physiologic scoring system (PRISM) is a valid predictor of length of stay;<br>3) The CRBSI is a preventable nosocomial infection that is financially costly and increases the PICU length of stay. Children using highly invasive technologies, such as CVVH and ECMO, have a much higher risk of CRBSI. Guidelines for the Prevention of Intravascular Devices in 2002 have been controversial because there is a limited amount of research on the benefits of some recommendations in paediatrics;<br>4) Loss of an airway by unplanned extubation can result in a longer length of ventilator dependence, a longer length of stay in the PICU, and increased risk of mortality; a nurse-driven sedation protocol would reduce this behaviour until the patient is ready to wean to extubate;<br>5) Medication administration guidelines are a central part of any kind of medical training, and yet, it remains the source of significant morbidity and mortality in hospitalized children; bedside nurses should rethink any double-checking protocol before a medication is administered
**Cummins et al, 2019** Indiana (U.S.), PICU	Intervention study (pre-post)	To implement evidence-based paediatric pressure injury prevention strategies to decrease the incidence of pressure injuries by reducing the rate from 8% to 6% in the PICU at a children's hospital in a large metropolitan city during a 6-week time period	Preintervention (n=197), Postintervention (n=560);<br>Nutrition consultation ordering for PICU patients at high risk for pressure injuries, Preintervention (n=33) Postintervention (n=31); aged <18 years; The PICU patient	Pressure ulcers (Prevalence and/or incidence rate)	(1) Educating PICU nurses on risk factors for paediatric pressure injuries and prevention strategies (2) turning PICU patients every 2 hours<br>(3) Ordering nutrition consultations on all patients with a Braden Q score less than 16	The PICU pressure injury incidence rate decreased from 8% to 3% during the quality improvement project, therefore the project aim was met. Patients with pressure injuries are at an increased risk of infection, experience pain from the pressure injuries, and stay in the hospital longer than patients without pressure injuries; the incidence of pressure injuries in PICU is 27%. Six paediatric pressure injury prevention interventions appeared consistently across several studies: (1) ensuring proper support surfaces for patients, (2) turning patients

			population is comprised of critically ill or injured children who have experienced trauma, severe infections, congenital anomalies, immunologic disorders, or have undergone extensive surgery			frequently, (3) ensuring proper nutrition for patients, (4) managing moisture for patients, (5) conducting routine skin assessments, and (6) the use of skin champions on units. All six intervention strategies were shown to reduce pressure injury incidence or prevalence throughout the studies. The Braden Q Scale is a validated and reliable tool used to predict paediatric pressure injury risk.
**Madelon et al, 2018** Minnesota (U.S.), PICU	Systematic review (Doctoral project)	To evaluate and synthesize evidence-based research that can be used to adapt a paediatric clinical guideline for sedation management	The analysis resulted in (n=17) articles meeting the relevant review criteria for the review selection procedure	Duration of sedation (Sedation days in the PICU)<br>Duration of mechanical ventilation (IMV-days)<br>LOS PICU (days)	Nurses' driven sedation protocol	Most studies (n = 9) resulted in better outcomes for patients, with the exception of one or three studies which found no significant differences. It was found that the use of nurses in the paediatric intensive care unit significantly reduced sedation days in the PICU. It is noted that the implementation of a nurse-led sedation protocol in the PICU has helped to reduce the duration of mechanical ventilation. There was a significant improvement in the length of stay in the PICU with protocol-directed sedation management. There is a significant difference in the direction of the length of stay and the days of mechanical ventilation. A significant improvement in sedation parameters was found with the implementation of the guidelines on sedation ($P < 0.001$) and it was found with the implementation and evaluation of a nurse-led paediatric sedation protocol in a PICU that the duration of mechanical ventilation was significantly shorter. However, no difference was found between the symptoms of discontinuing sedation. Lack of RCTs was noted as limited factors in multiple studies
**Kline et al, 2005** Illinois (U.S.), PICU	Narrative review	To show the strategies present in the literature for the prevention of CRBSI	NR	CRBSI (CRBSI per 1,000 catheter days)	NR	Strategies to Reduce Risk of Catheter-related Bloodstream Infections:<br>1) Site selection for catheter placement; in children, femoral catheters have demonstrated a lower incidence of mechanical complication and may have an equivalent incidence of infection than catheters in alternate sites;<br>2) Both the material of the catheter and the intrinsic virulence factors of the offending organism affect the pathogenesis of CRBSI; In vitro studies have demonstrated that microorganisms are more adherent to catheters

produced of polyvinyl chloride or polyethylene than catheters composed of Teflon, silicon elastomer, or polyurethane;
3) Hand hygiene contributes significantly to CRBSI risk;
4)The CDC recommendations incorporate maintaining aseptic technique for the insertion and care of intravascular catheters.
5) Central catheter site dressing changes should be performed utilizing sterile gauze or sterile transparent semipermeable dressing to cover the catheter site;
6) Certain antibiotic or antiseptic-impregnated catheters can reduce the risk for CRBSI;
7) No relationship was found between duration of catheterization and the daily probability of infection, suggesting that routine replacement of central venous catheters likely does not reduce the incidence of CRBSI

Abbreviations: PICU, Pediatric intensive care unit; ICU, Intensive care unit; MICU, Medical intensive care unit; SICU, Surgical intensive care unit; PCICU, Pediatric cardiac intensive care unit; LOS, Hospital length of stay; VAP, Ventilator associated pneumonia; CRBSI, Catheter-related bloodstream infection; CAUTI, Catheter Associated Urinary Tract Infections; CLABSI, Central line-associated bloodstream infection; PRISM3, pediatric risk of mortality score3; PIM3, Pediatric index of mortality score 3; PIM2, Pediatric Indicator of Mortality 2 NR, non-reported; IMV, Invasive mechanical ventilation; DOV, Durations of invasive mechanical ventilation; TVT, Total ventilation time; WD, Weaning duration; VFDs, Ventilator-free days; VAFDs, vasoactive-free days; PEWS, Pediatric Early Warning Systems; CDR PEWS, Central Denmark Region PEWS; SOFA, Sequential Organ Failure Assessment; GCS, Glasgow Come scale; FOUR, Full Out line of Un Responsiveness; RBC, Red Blood Cells; PPFT, Post-pyloric feeding tubes; ICP, Intracranial pressure; MAP, Mean arterial pressure; CPP, Cerebral perfusion pressure; PIVC, Peripheral Intravenous Catheters; PN, Parenteral nutrition; EN, Enteral nutrition; NST, Nutrition support team; GRV, Gastric residual volume; OM, Oral mucositis; OCG, Oral care guide; OAS, Oral Assessment Scale; CINC, Continuity in nursing care; CCI, Continuity of Care Index; ETSMV, Endotracheal suctioning and manual ventilation; LR, Log-rolling; VAE, Ventilator-associated events; VAC, Ventilator-associated condition; CDC, Centers for Disease Control; NPMODS, new or progressive multiple organ dysfunction syndrome; CAPD, Cornell Assessment of Pediatric Delirium; pCAM-ICU, Confusion Assessment Method for the Intensive Care Unit; PELOD, Pediatric Logistic Organ Dysfunction score; PEWS, Pediatric Early Warning Systems; ECMO, Extra Corporal Membrane Oxygenation; CPAP, Continuous Positive Airway Pressure; OT, Occupational therapist; PT, Physical therapist; CBS, Comfort behavior scale; U.S.A., United State of America.
